# Mapping the perception-space of facial expressions in the era of face masks

**DOI:** 10.3389/fpsyg.2022.956832

**Published:** 2022-09-13

**Authors:** Alessia Verroca, Chiara Maria de Rienzo, Filippo Gambarota, Paola Sessa

**Affiliations:** ^1^Department of Developmental and Social Psychology, University of Padova, Padova, Italy; ^2^Padova Neuroscience Center (PNC), University of Padova, Padova, Italy

**Keywords:** emotion recognition, alexithymia, autistic traits, COVID-19, face mask, facial expressions

## Abstract

With the advent of the severe acute respiratory syndrome-Corona Virus type 2 (SARS-CoV-2) pandemic, the theme of emotion recognition from facial expressions has become highly relevant due to the widespread use of face masks as one of the main devices imposed to counter the spread of the virus. Unsurprisingly, several studies published in the last 2 years have shown that accuracy in the recognition of basic emotions expressed by faces wearing masks is reduced. However, less is known about the impact that wearing face masks has on the ability to recognize emotions from subtle expressions. Furthermore, even less is known regarding the role of interindividual differences (such as alexithymic and autistic traits) in emotion processing. This study investigated the perception of all the six basic emotions (anger, disgust, fear, happiness, sadness, and surprise), both as a function of the face mask and as a function of the facial expressions’ intensity (full vs. subtle) in terms of participants’ uncertainty in their responses, misattribution errors, and perceived intensity. The experiment was conducted online on a large sample of participants (*N* = 129). Participants completed the 20-item Toronto Alexithymia Scale and the Autistic Spectrum Quotient and then performed an emotion-recognition task that involved face stimuli wearing a mask or not, and displaying full or subtle expressions. Each face stimulus was presented alongside the Geneva Emotion Wheel (GEW), and participants had to indicate what emotion they believed the other person was feeling and its intensity using the GEW. For each combination of our variables, we computed the indices of ‘uncertainty’ (i.e., the spread of responses around the correct emotion category), ‘bias’ (i.e., the systematic errors in recognition), and ‘perceived intensity’ (i.e., the distance from the center of the GEW). We found that face masks increase uncertainty for all facial expressions of emotion, except for fear when intense, and that disgust was systematically confused with anger (i.e., response bias). Furthermore, when faces were covered by the mask, all the emotions were perceived as less intense, and this was particularly evident for subtle expressions. Finally, we did not find any evidence of a relationship between these indices and alexithymic/autistic traits.

## Introduction

Communicating one’s emotions and recognizing the emotions of others are crucial skills that allow an understanding of other people’s affective states and intentions and help build/foster interpersonal relationships.

In this respect, humans, along with other primates, have developed a complex facial musculature that allows a rich variety of configurations, thereby enabling them to convey a multitude of possible emotions: muscles distributed in different areas of the face contribute to the production of different expressions. Conversely, an observer will use the visual information distributed over another person’s face to recognize the emotion being expressed. Thus, it is clear that any circumstance that prevents a person from seeing another person’s entire face will also reduce the degree of correct recognition of that person’s expression and, therefore, of their emotion.

The advent of the COVID-19 pandemic and the introduction of face masks as a protective device to limit the spread of the infection has raised considerable interest in the context of studies on face processing, as masks made it impossible to view the entire lower half of the face. Several studies conducted after the beginning of the pandemic (and the use of face masks) have investigated potential patterns in the recognition of emotions by comparing conditions in which the faces were entirely visible with conditions in which the faces were covered by a mask (Carbon, 2020; Grundmann et al., 2020; Ruba and Pollak, 2020; Bani et al., 2021; Calbi et al., 2021; Carbon and Serrano, 2021; Fitousi et al., 2021; Gori et al., 2021; Grahlow et al., 2021; Kang et al., 2021; Lau, 2021; Marini et al., 2021; Noyes et al., 2021; Pazhoohi et al., 2021; Sheldon et al., 2021; Ziccardi et al., 2021; Carbon et al., 2022; Grenville and Dwyer, 2022; Kastendieck et al., 2022; Kim et al., 2022; Langbehn et al., 2022; Maiorana et al., 2022; McCrackin et al., 2022; Parada-Fernández et al., 2022; Schneider et al., 2022; Tsantani et al., 2022). Previous studies had also investigated the ability to extract affective meaning from only partially visible faces, using different occlusion methods such as the following: presenting stimuli covered by hats, scarves, sunglasses, niqabs, or censoring black bars; degrading the quality of sections of the presented image; or progressively increasing the visual information available (Kret and de Gelder, 2012; Calvo and Fernández-Martín, 2013; Calvo et al., 2014; Wegrzyn et al., 2017; Kret and Fischer, 2018; Liedtke et al., 2018; Ruba and Pollak, 2020; Kret et al., 2021; Noyes et al., 2021; Kim et al., 2022). Overall, these studies found that the use of facial masks and other occlusion methods does interfere with the ability to accurately recognize facial expressions of emotion but not to the extent that it is reduced to chance level (Pavlova and Sokolov, 2022).

It should be noted that the degree of such interference has been found to vary according to the different emotions expressed. For example, it appears that the recognition of anger is not always affected by the occlusion caused by face masks. This would seem to indicate that access to the visual information conveyed by the upper portion of the face is sufficient for its correct recognition (Carbon and Serrano, 2021; Grenville and Dwyer, 2022; Schneider et al., 2022; Tsantani et al., 2022). Surprisingly, it has been observed how anger can be easier to identify when the lower part of the face is covered (Carbon and Serrano, 2021; Ziccardi et al., 2021; Grenville and Dwyer, 2022). The correct recognition of fear has also been found unnecessarily hindered by the use of face masks (Carbon, 2020; Ruba and Pollak, 2020; Bani et al., 2021; Carbon and Serrano, 2021; Pazhoohi et al., 2021; Carbon et al., 2022; Grenville and Dwyer, 2022). Recognition of the expressions of happiness, disgust, and sadness, on the other hand, tends to be particularly compromised when the face is covered. The misattribution errors that have been observed include confusing happiness with surprise (Ziccardi et al., 2021), anger with sadness (Kim et al., 2022; Schneider et al., 2022), disgust with anger (Carbon, 2020; Ziccardi et al., 2021; Carbon et al., 2022; Kim et al., 2022; Tsantani et al., 2022) and sadness (Carbon and Serrano, 2021; Ziccardi et al., 2021; Kim et al., 2022), and sadness with disgust (Carbon, 2020; Carbon et al., 2022; Kim et al., 2022), anger (Carbon et al., 2022; Kim et al., 2022; Schneider et al., 2022), and fear (Carbon, 2020; Carbon et al., 2022). Interestingly, it has also been found that the presence of face masks can result in observers mistaking happy expressions for neutral ones, erroneously considering the latter as expressions of sadness (Marini et al., 2021).

So far, almost all of the studies conducted have investigated the perception and recognition of intense facial expressions using static images as stimuli (Carbon, 2020; Grundmann et al., 2020; Ruba and Pollak, 2020; Calbi et al., 2021; Carbon and Serrano, 2021; Fitousi et al., 2021; Grahlow et al., 2021; Kang et al., 2021; Lau, 2021; Marini et al., 2021; Noyes et al., 2021; Pazhoohi et al., 2021; Ziccardi et al., 2021; Carbon et al., 2022; Grenville and Dwyer, 2022; Kastendieck et al., 2022; Kim et al., 2022; Langbehn et al., 2022; Maiorana et al., 2022; McCrackin et al., 2022; Parada-Fernández et al., 2022; Schneider et al., 2022; Tsantani et al., 2022). The few exceptions to this have investigated the recognition of subtle (Bani et al., 2021; Gori et al., 2021; Sheldon et al., 2021) and ambiguous/blended (Wegrzyn et al., 2015) expressions using static pictures or short video clips as dynamic stimuli (Kastendieck et al., 2022; Langbehn et al., 2022). In most cases, the datasets used by the studies conducted so far have presented photos of actors reproducing specific emotions in an exaggerated and prototypical manner. The images selected to represent the target emotions that participants were asked to identify often portrayed expressions of an intense emotional activation, leaving little to no space for the representation of more subtle degrees of intensity. This choice of stimuli has led to an unnatural representation of reality since in everyday life, nature constantly presents us with subtly nuanced expressions of emotion. Addressing this issue is of great importance since recognizing subtly nuanced emotions may prove more arduous than recognizing the same emotions expressed in a more exaggerated manner, making it harder to avoid errors of judgment that were previously not observed. For this reason, in the present study, we decided to present stimuli showing both facial expressions portraying emotions felt very intensely and facial expressions representing emotions felt less strongly. To our knowledge, only three studies have specifically investigated the recognition of subtle expressions in adult faces covered by face masks. The first, conducted by Sheldon et al. (2021), studied the perception of different types of smiles (Duchenne and social), particularly demonstrating that the presence of face masks tends to reduce the perception of the social smile’s pleasantness. The second study by Bani et al. (2021), on the other hand, investigated the ability of young medical and nursing students to recognize four basic emotions (fear, happiness, sadness, and anger) presented at different intensity levels, both with and without a face mask. The results of this study supported previous evidence by demonstrating an impaired recognition accuracy in the masked condition. Furthermore, with the exception of fear, different intensity levels in the masked condition produced a greater proportion/a higher number of emotion misattribution errors than were observed in the condition of complete facial visibility. The third study that was conducted by Gori et al. (2021) examined this topic from a developmental perspective, finding the use of face masks to have a negative impact on the ability of toddlers, children, and adults to infer emotions from masked facial configurations expressing happiness, fear, anger, and sadness or portraying a neutral expression. Moreover, the study found toddlers’ performances to be the most affected by the presence of face masks when compared to those of both children and adults.

It has been observed that the presence of masks tends to impact the perception of an emotion’s intensity. More specifically, when covered by masks, facial expressions tend to be perceived as more subdued. It has been observed that the same facial expression has been judged to convey an emotion less intensely when covered (Pazhoohi et al., 2021; Kastendieck et al., 2022; Tsantani et al., 2022). It has also been observed that some specific emotions seem to be more affected by this than others and that the perception of the intensity of happiness appears to be particularly compromised (Sheldon et al., 2021; Langbehn et al., 2022; Ramachandra and Longacre, 2022). Another interesting aspect is that not only does the intensity of target emotions displayed behind masks appear to be reduced, but also when asked to indicate whether other distractor emotions are perceived as present in the image, participants tend to indicate these as more present in faces covered by masks than in fully visible faces (e.g., Tsantani et al., 2022).

The lack of in-depth knowledge regarding how the presence of face masks affects the perception of emotions’ intensity may be due to the fact that the majority of studies conducted so far have employed tasks requiring participants to assess a person’s emotional state from a limited list of given emotions (Kret and Fischer, 2018; Liedtke et al., 2018; Carbon, 2020; Grundmann et al., 2020; Ruba and Pollak, 2020; Bani et al., 2021; Calbi et al., 2021; Carbon and Serrano, 2021; Gori et al., 2021; Grahlow et al., 2021; Kang et al., 2021; Marini et al., 2021; Noyes et al., 2021; Pazhoohi et al., 2021; Ziccardi et al., 2021; Carbon et al., 2022; Grenville and Dwyer, 2022; Kim et al., 2022; Maiorana et al., 2022; McCrackin et al., 2022; Parada-Fernández et al., 2022; Schneider et al., 2022). Although such methods allow one to observe whether participants confuse the facial expressions of one emotion with another unintended one, they do not provide any information regarding the perception of the intensity of the emotion identified. In this study, we therefore decided to ask participants to provide us with this information by using the Geneva Emotion Wheel (GEW; Scherer, 2005; Scherer et al., 2013) to indicate their perception of the emotional intensity expressed by the target stimuli.

To date, most published studies have chosen to select stimuli representing only a limited variety of emotions (a range of three to four emotions) (Kret and Fischer, 2018; Liedtke et al., 2018; Ruba and Pollak, 2020; Bani et al., 2021; Calbi et al., 2021; Fitousi et al., 2021; Lau, 2021; Marini et al., 2021; Kastendieck et al., 2022; Maiorana et al., 2022; Parada-Fernández et al., 2022; Schneider et al., 2022). In the present study, we therefore decided to assess recognition of all the six basic emotions. Interestingly, the valence of the emotions selected in these previous studies has not always been evenly represented. For example, in some studies, happiness has been the only emotion with positive valence (Kret and Fischer, 2018; Liedtke et al., 2018; Bani et al., 2021; Marini et al., 2021; Maiorana et al., 2022; Schneider et al., 2022). In experiments presenting participants with a forced-choice task, this has occasionally created a real risk of registering a ceiling effect, as observed in Kastendieck et al. (2022). Moreover, the number of studies using stimuli representing a range of emotions presented with varying degrees of intensity is, at present, very restricted (Bani et al., 2021; Gori et al., 2021; Sheldon et al., 2021). As a result, the recognition of certain emotions (such as disgust or surprise) presented in subtle expressions and partly hidden by masks has seldom been studied.

Finally, very few studies have investigated the associations between the presence of clinical traits and difficulties in recognizing facial expressions of emotions covered by masks (Calbi et al., 2021; Pazhoohi et al., 2021; Ziccardi et al., 2021; Maiorana et al., 2022; McCrackin et al., 2022; Ramachandra and Longacre, 2022). Many studies have, however, investigated normal-typical subjects’ behavior both in relation to traits associated with social affiliation (Calbi et al., 2021), empathy (Liedtke et al., 2018; Calbi et al., 2021; Trainin and Yeshurun, 2021; Carbon et al., 2022; McCrackin et al., 2022; Ramachandra and Longacre, 2022) and in relation to personal impressions regarding the COVID-19 pandemic (Grundmann et al., 2020; Calbi et al., 2021; Trainin and Yeshurun, 2021; Carbon et al., 2022; Tsantani et al., 2022). Autism and alexithymia traits are hypothesized to be among the clinical traits thought to be particularly affected by the presence of facial masks. Not all of the studies that have submitted questionnaires investigating the presence of alexithymic traits (Calbi et al., 2021; Maiorana et al., 2022) have used the gathered data to analyze the relationship between the presence of such traits and participants’ performance in emotion-recognition tasks, showing only the upper part of the face (see Calbi et al., 2021). The limited sample sizes analyzed in studies examining the presence of autistic traits in participants performing facial-expressions recognition tasks (Pazhoohi et al., 2021; McCrackin et al., 2022; Ramachandra and Longacre, 2022), have often prevented them from reaching clear-cut conclusions. Furthermore, these studies have frequently presented participants with stimuli showing only cut-away sections of the human face expressing the target emotion (for example, the eyes) rather than specifically addressing the issue of facial-expression recognition in the presence of face masks.

The present study aimed to clarify how the perception of all the six basic emotions (anger, disgust, fear, happiness, sadness, and surprise) varies both according to whether the face observed has been covered by a face mask and according to the intensity of the facial expression represented (full−100% vs. subtle−40%). We also explored whether individuals’ autistic and alexithymic traits may have an impact on facial-expression recognition.

**FIGURE 1:**
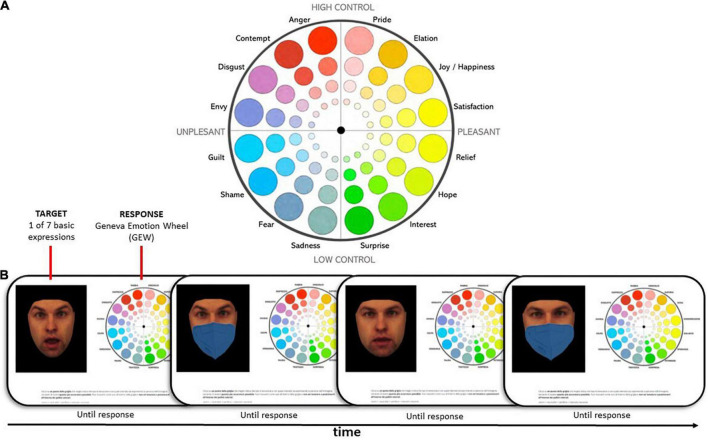
Description of the experimental paradigm. **(A)** English version of the GEW 1.0. During the experiment, the labels were presented in Italian. **(B)** Trial example. Each trial consisted of a screen with the target face with or without the facial mask. The facial expression intensity could be full or subtle (except for neutral faces). Next to the face, the GEW was used to collect the response. There was no time limit, and the next trial start was self-paced. Images reproduced from KDEF (Lundqvist et al., 1998) with permission. Identity AM02 from the KDEF image set is depicted.

We used the GEW (Scherer, 2005; Scherer et al., 2013) to collect responses from participants. The GEW is an instrument designed to combine both a discrete and a dimensional approach in the self-report assessment of emotion (see the upper panel of Figure 1). We chose this tool because it allowed us to evaluate the participants’ performance for all six basic emotions by concurrently reducing the probability of ceiling effects. It also allowed us to measure three different indices based on participants’ single responses (i.e., single clicks on the wheel): (1) uncertainty in their responses (the spread around the angle of the correct emotion category segment), i.e., the tendency to confuse (not necessarily in a systematically direction) one emotion with others; (2) response bias (the mean angle of deviation from the angle of the correct emotion category segment), i.e., the systematic tendency to confuse one emotion with another emotion/other emotions by systematically choosing emotion categories positioned clockwise or anticlockwise in the GEW; and (3) perceived intensity (the mean distance from the center of the GEW).

Since the presence of face masks reduces the available information helping a person to decide which emotion is being expressed by a face, we expected—on the basis that information can be conceived as a reduction of uncertainty—to find that the manipulation involving the presence or absence of a mask would have an impact on the uncertainty index for *all facial expressions*. That is to say, we hypothesized an increased uncertainty in the participants’ responses relating to facial expressions covered by a mask, compared to those relating to uncovered facial expressions. If this is the case, this effect would offer an index for quantifying more precisely the impact of face masks on participants’ confidence in their judgment, which is reduced as a function of face masks (Carbon, 2020). We further expected a greater increase in uncertainty for subtle facial expressions since they provide even less overall available information when covered by face masks. Nevertheless, we envisaged that facial expressions characterized by highly distinctive modifications in the upper portion of the face could be immune to this reduction of information, especially when intense.

Applying Action Units (AUs; Ekman and Friesen, 1978^1^) as diagnostic information for the correct recognition of emotional expressions, the basic emotions conveyed primarily by the lower portion of the face are *disgust* (characterized by wrinkling of the nose and lifting of the upper lip) and *happiness* (mainly characterized by the raising of the corners of the mouth). With regard to disgust, the only additional secondary AU possibly available when the face is covered is AU7 (tension of the inferior eyelid). It should be noted that the only expression distinctively characterized by AU7 is anger. Therefore, it may be hypothesized that, when observing a face covered by a mask, the expression of disgust may be confused with anger. On this basis, we expected to observe a response bias toward anger in cases where expressions of disgust were subject to face-mask manipulation. With regard to happiness, there are many additional AUs that can be activated, so we did not make any specific hypotheses about whether the presence of face masks could impact the response bias.

We expected to replicate previous results regarding the degree of intensity perceived as a function of the mask (e.g., Pazhoohi et al., 2021; Kastendieck et al., 2022; Tsantani et al., 2022) and, therefore, to observe a reduction in the perceived intensity of the expressions covered by face masks when compared to uncovered expressions. Furthermore, we expected to find this effect to be particularly evident for subtle expressions, considering they could be more easily misinterpreted as neutral expressions.

Finally, we expected to find that all the hypothesized effects described above would also correlate with alexithymic and autistic traits assessed by means of the 20-item Toronto Alexithymia Scale (20-item TAS; Bagby et al., 1994) and the Autism Quotient (AQ; Baron-Cohen et al., 2001). We expected to observe a relationship between these traits and the three indices described above.

## Materials and methods

### Participants

We recruited a total of 139 volunteers to participate in this study. The data obtained from 10 participants were not included in our analyses because the level of accuracy of the responses registered in the catch trials was not deemed sufficient, which is <75% accuracy. We arbitrarily decided that performance above 75% accuracy on catch trials was sufficient to ensure that the participants had focused on the main task. Thus, our final sample size consisted of 129 participants, of whom 116 were women (M_age_ = 23.3, SD = 2.99) and 13 were men (M_age_ = 26.4, SD = 6.45).

All participants were Italian native speakers of Italian nationality to avoid registering possible differences linked to the culture of origin of those participating. To ensure a correct and homologous vision of the stimuli, participants were explicitly requested to perform the test from their personal computers only, after having duly calibrated their screen following specific instructions; this allowed everyone to view both the images of the target stimuli and the GEW in the same dimensions (8 cm width for the target stimuli and 300 × 300 pixels for the GEW). The study was created to be administered online, and volunteers were mainly recruited *via* announcements posted on social networks. The majority of the sample comprised students from the University of Padua. All volunteers provided informed consent before participating in the study.

### Materials

#### Questionnaires

Participants were asked to complete a questionnaire providing their demographic data and contact information. They also completed the TAS-20 questionnaire to investigate the presence of alexithymic traits (Bagby et al., 1994) and the AQ questionnaire to investigate the presence of autistic traits (Baron-Cohen et al., 2001).

#### Stimuli

A total of 260 experimental stimuli and 14 catch trials were administered during the test phase, which was preceded by a familiarization phase involving 10 stimuli and 1 catch trial. Seventy face stimuli were selected from the Karolinska Directed Emotional Faces database (KDEF^2^; Lundqvist et al., 1998), for a total of 10 Caucasian identities (5 female and 5 male face stimuli; AF05, AF06, AF07, AF08, AF09, AM01, AM01, AM02, AM03, AM04, AM05, and AM06) portraying the 6 basic emotions (anger, disgust, fear, happiness, sadness, and surprise) and the neutral expression.

The images were modified with a Face Morpher script^3^ by morphing each of the six emotional expressions with the neutral expression to obtain a realistic facial conformation with an intensity of the expressed emotion equal to 40%. This allowed us to obtain full and subtle expressions of each of the six basic emotions. Given that the background of each image was removed and replaced by a black backdrop, each stimulus consisted solely of a face portraying the target emotion. Each stimulus was then duplicated and an N95 mask was affixed to each copy, using the MaskTheFace script^4^. As a result, each facial expression was represented by an unmasked and a masked face expressing both a full and a subtle manifestation of intensity. Each catch trial consisted of an image of the same size as those containing faces, with a sentence written in white color (in Italian) on a black background asking the participant to click on a specific position of the Geneva Emotion Wheel. The request was different for each of the 14 catch trials, and they were randomly presented during the experimental phase (7 per block). Each facial stimulus was presented to participants only once, and each participant saw all of the ten identities with block randomization across subjects. Trails were randomized within each block but they were not randomized between blocks. To summarize, we administered 240 emotional faces (60 masked/100% intensity, 60 masked/40% intensity, 60 unmasked/100% intensity, and 60 unmasked/40% intensity), plus 20 neutral faces (10 masked and 10 unmasked), plus 14 catch trials, for a total of 274 trials.

#### Geneva emotion wheel

We used the Geneva Emotion Wheel (GEW 1.0; Tran, 2004; Vaughan, 2011) to gather the participants’ responses. The terms used to refer to the emotions included in the GEW were translated into Italian by bilingual English/Italian speakers. The GEW 1.0 presents 16 terms indicating different emotions arranged around the wheel’s circumference. Each emotion is represented by a series of 4 differently sized circles proceeding outward from the center of the circle, with their size corresponding to the increasing intensity of the emotion perceived. The center of the wheel thus represents a point of neutrality. The emotions are distributed according to the degree of control/power (low at the bottom of the GEW and high at the top) and the valence (with the more negative emotions arranged on the left and the more positive emotions arranged on the right). The GEW was originally designed to allow participants to indicate their experienced emotions as precisely as possible, but it has also been used on several occasions to indicate perceived emotions in others (e.g., Siegert et al., 2011; Zheng and Xia, 2012; Coyne et al., 2020). This tool seeks to represent emotions both discretely and continuously; emotions that partially share the same characteristics of control and valence are placed in proximity but constitute distinct radii; emotions that possess opposite characteristics are placed diametrically opposite to each other. We decided to use the GEW and, specifically, version 1.0 (Tran, 2004; Vaughan, 2011), which includes 16 emotions (plus the neutral condition) and 4 degrees of intensity, for the following reasons: (i) it is easy for participants to use; (ii) its use avoids ceiling effects because participants have to choose the correct emotion from among a series of distractors; and (iii) because the latest 3.0 version does not include the basic emotion of “surprise.”

### Procedure

We used the Gorilla Experiment Builder^5^ to create and host our experiment (Anwyl-Irvine et al., 2020). The experiment lasted about 40 min and was carried out using a computer online. After giving their consent and completing the TAS-20 and AQ questionnaires, participants began the experimental session. They received the experimental instructions, were familiarized with the GEW, and performed a series of test trials. More specifically, participants were told that they could click anywhere on the wheel, including outside the circles if they felt it was appropriate. The face stimulus, the GEW, and a reminder of the instructions were simultaneously presented during each trial (see Figure 1; identity AM02 from the KDEF is depicted in Figure 1). The session was divided into two randomized blocks each comprising 137 trials and lasting approximately 15 min. The two blocks were separated by a pause, the length of which was decided by each participant. Full, subtle, and neutral emotions were randomly presented in each block. Each emotion was represented by different models and each model represented all the emotions that were presented as stimuli. The stimuli were presented without a time limit and the subsequent trial started immediately after the participant had given a response by clicking on the GEW. Only one response was accepted for each trial. Catch trials were distributed throughout the experiment to ensure that participants were vigilant while providing their answers.

### Data analysis

To assess participants’ perception of the facial expressions they were presented with, we transformed their responses into polar coordinates. The Euclidean distance from the GEW center (measured in pixels) indicates the *perceived intensity* of the emotion evaluated. Facial expressions perceived as more intense are represented by a greater distance. The *angle*, measured in degrees, corresponds to the response orientation around the GEW. We created a measure of participants’ performance by computing the difference between the *response* angle (in radians) and the angle of the presented emotion (i.e., the *correct* angle). The correct angle was computed by dividing the GEW into equal parts and then centering each emotion. For better interpretability, we transformed the angles into degrees. In this way, we centered participants’ responses on the displayed emotion with errors that ranged between −180° and 180°. Values around 0 mean correct responses while negative and positive values represent, respectively, an anticlockwise and clockwise shift of responses on the wheel.

This measure allowed us to extract two important indices. The circular mean of responses representing the average direction on the circle, relating to the displayed emotion, constitutes the *bias*. When the *bias* is different from zero, there is evidence of a systematic response shift to another location on the GEW.

The circular variability constitutes the *uncertainty* and, independently of the bias, it provides information about the amount of spread in participants’ responses. For example, an emotion could be systematically confused with other emotions (i.e., *bias* different from 0), but this misattribution may be characterized by a low level of *uncertainty*, or, in another scenario, there could be no systematic *bias* toward a specific direction but a high-response *uncertainty* (i.e., greater circular variance).

Given the shape of the GEW, to analyze our data, standard statistical models were not appropriate (Cremers and Klugkist, 2018). Therefore, we decided to use a generalized linear mixed model, using the Von Mises distribution as the likelihood function to model both the *bias* and the *uncertainty*. The Von Mises is the circular version of the Gaussian distribution where parameters (μ and *k*) are directly associated with our *bias* and *uncertainty* indices. The parameter μ (the circular mean) represents the *bias* and the *k* parameter (the Von Mises *concentration*) represents the *uncertainty*. To facilitate interpretation, we transformed *k* into circular variance (Evans et al., 2011, 191–192). With this transformation, *k* values are bounded between 0 (i.e., all values are concentrated on a single point, minimum *uncertainty*) and 1 (i.e., values are uniform around the circle, maximum *uncertainty*). Given the relevance and the independence of *bias* and *uncertainty*, we decided to analyze both aspects in the same model. Using the so-called location-scale modeling (Rigby and Stasinopoulos, 2005; Bürkner, 2018), both μ (*bias*) and *k* (*uncertainty*) can be predicted within the same model. To model the *perceived intensity*, we used a standard general linear mixed-effects model. In dealing with the multilevel data structure, we added the participants’ random effect in each model.

As predictors, we used *Mask* (faces with and without the facial mask), *Facial Expression Intensity* (full and subtle), and the displayed *Emotion* (anger, happiness, fear, surprise, disgust, and sadness).

As an exploratory analysis, we also analyzed the impact of alexithymia and autistic traits using the TAS and the AQ questionnaire. In this case, we fitted a model with the interaction between Mask and TAS/AQ for *bias*, *uncertainty*, and *perceived intensity*. Given that the Mask effect could be different according to the Facial Expression Intensity, we fitted the same model considering only the subtle facial expressions.

We calculated all models under Bayesian framework. Bayesian statistics combine previous knowledge (i.e., *priors*) with empirical data (i.e., the *likelihood*) to compute the *posterior* probability. We decided to use a Bayesian approach for several reasons. Firstly, compared to the frequentist approach, each parameter in a Bayesian regression model is represented by a probability distribution of plausible values after combining data with prior knowledge, instead of a single estimated value (Kruschke and Liddell, 2018). Secondly, the Bayesian framework allows more modeling flexibility and reliability for complex models (Bolker et al., 2009). To our knowledge, the location-scale Von Mises regression can be easily implemented only within a Bayesian framework.

For the model fitting, we used the *brms* package (Bürkner, 2017, 2018) based on the STAN probabilistic programming language (Stan Development Team, 2022) and R (R Core Team, 2022). We decided to use weakly informative priors for regression parameters (Gelman, 2006; Gelman et al., 2017). These priors allow more modeling efficiency by excluding very implausible or impossible values. In this way, posterior distributions are mainly influenced by the data (i.e., *likelihood*). All models converged according to the Gelman and Rubin (1992) R̂ value. Details of the Models, the priors’ specifications, and the diagnostics are available in the Supplementary material and the online OSF repository^6^.

For each response variable, we used the following analytical approach. We tested the Mask effect (Δ Mask = Mask_yes_−Mask_no_) for each displayed emotion. This allowed us to directly assess the impact of the facial mask on facial-expression perception in terms of *bias* and *perceived intensity*. For the *uncertainty*, the Mask effect is computed using the ratio between conditions (Ratio Mask = Mask_yes_/Mask_no_), as commonly used for variance-like measures (Nakagawa et al., 2015).

Next, we tested whether the Mask effect differs when considering *subtle* or *full* facial expressions. First, we compared the model with and without the three-way interaction among Mask, Emotion, and Facial-expression Intensity using the Pareto-Smoothed Importance Sampling Leave-One-Out cross-validation criterion (PSIS-LOO). The PSIS-LOO is a more robust variant of the WAIC index (i.e., the Bayesian alternative to the Akaike Information Criterion) that can be used for model comparisons (Vehtari et al., 2017). In this way, we can assign a probability value to both models and find the most plausible. Then, we calculated the Intensity effect as the difference between Mask deltas for *subtle* and *full* facial expressions (Δ Intensity = Δ Mask_full_−Δ Mask_subtle_) in relation to the *bias* and the *perceived intensity*. For the *uncertainty*, we calculated the ratio between Mask ratios (Ratio Intensity = Ratio Mask_full_/Ratio Mask_subtle_).

We summarized each model parameter or posteriors contrast using the median and the 95% Highest Posterior Density Interval (HPDI). The 95% HPDI is the interval of the posterior distribution that contains 95% of the most plausible values (Kruschke and Liddell, 2018). We consider a result as statistically significant if the null value, e.g., 0 is not contained within the 95% HPDI. For the *perceived intensity* and *bias*, each relevant contrast (i.e., difference) is bidirectionally tested against 0, whereas for the *uncertainty*, we tested the contrasts (i.e., ratio) against 1. If possible, we reported the Bayes Factor calculated using the Savage-Dickey density ratio (Wagenmakers et al., 2010) to support evidence for the null effect.

## Results

**FIGURE 2:**
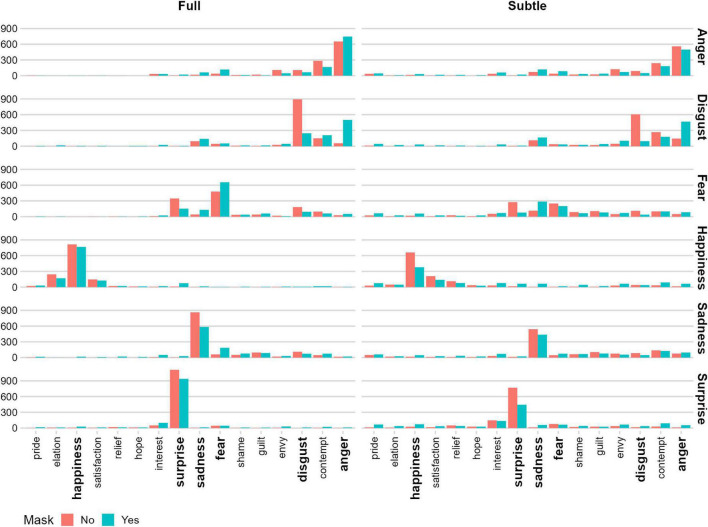
GEW responses as a function of displayed Emotion, Mask, and Facial Expression Intensity. Each participant’s response was classified according to the position on the GEW to assign a label. The order of the *X*-axis corresponds to the clockwise order of the GEW. The *Y*-axis represents relative frequencies in each condition.

Participants’ responses as a function of Mask, Facial-Expression Intensity, and Emotion, expressed through the GEW location, are depicted in Figure 2.

### Bias

The first model predicts the *bias* with Mask, Emotion, and Facial-Expression Intensity as predictors. Posterior distribution summaries for the Mask effect and the interaction between Mask and Facial-Expression Intensity are presented in Tables 1, 2, respectively.

**TABLE 1:** Posterior distribution summaries for the *bias* and *uncertainty* Mask effect as a function of the displayed Emotion.

Emotion	Parameter	Mask_*yes*_	Mask_*no*_	Contrast
Surprise	Bias	−0.576 [−3.997, 2.751]	−0.846 [−2.216, 0.525]	0.28 [−3.342, 3.829]
Sadness		24.284 [20.466, 28]*	29.292 [26.641, 32.021]*	−4.972 [−9.738, −0.548]*
Happiness		2.553 [−0.427, 5.581]	2.834 [1.496, 4.167]*	−0.276 [−3.609, 2.855]
Fear		6.28 [2.779, 9.595]*	5.463 [2.789, 8.159]*	0.835 [−3.548, 5.121]
Disgust		17.735 [15.205, 20.43]*	−0.657 [−2.162, 0.859]	18.392 [15.403, 21.321]*
Anger		−22.361 [−25.222, −19.563] *	−20.244 [−22.062, −18.403] *	−2.125 [−5.526, 1.132]
Surprise	Uncertainty	0.441 [0.419, 0.464]*	0.166 [0.152, 0.179]*	2.818 [2.608, 3.033]*
Sadness		0.533 [0.507, 0.559]*	0.415 [0.392, 0.439]*	1.324 [1.237, 1.415]*
Happiness		0.418 [0.395, 0.44]*	0.156 [0.144, 0.169]*	2.842 [2.635, 3.068]*
Fear		0.481 [0.457, 0.507]*	0.467 [0.439, 0.493]*	0.997 [0.935, 1.061]
Disgust		0.449 [0.423, 0.475]*	0.203 [0.187, 0.219]*	2.229 [2.064, 2.401]*
Anger		0.474 [0.447, 0.5]*	0.278 [0.258, 0.298]*	1.714 [1.592, 1.836]*

Distributions are summarized using the median and 95% HPDI. Asterisks represent contrasts where the null value (i.e., 0 for deltas or 1 for ratios) is not contained in the 95% HPDI.

**TABLE 2:** Posterior distribution summaries for the *bias* and *uncertainty* Facial Expression Intensity effect as a function of the displayed Emotion.

Emotion	Parameter	Δ *Mask*_full_	Δ *Mask*_subtle_	Contrast
Surprise	Bias	0.031 [−2.378, 2.36]	0.513 [−6.448, 7.235]	−0.461 [−8.005, 6.513]
Sadness		1.572 [−2.076, 5.417]	−11.512 [−20.061, −3.229]*	13.09 [3.846, 22.37]*
Happiness		3.473 [1.155, 5.784]*	−4.026 [−10.139, 1.936]	7.496 [1.117, 14.079]*
Fear		−0.981 [−5.034, 3.2]	2.617 [−4.97, 10.195]	−3.595 [−12.151, 5.129]
Disgust		19.08 [15.563, 22.79]*	17.692 [12.967, 22.375]*	1.393 [−4.409, 7.449]
Anger		−0.032 [−3.703, 3.827]	−4.212 [−9.673, 1.349]	4.171 [−2.498, 10.848]
Surprise	Uncertainty	3.073 [2.74, 3.433]*	2.559 [2.303, 2.81]*	1.2 [1.026, 1.387]*
Sadness		1.425 [1.281, 1.583]*	1.221 [1.13, 1.317]*	1.166 [1.022, 1.325]*
Happiness		3.128 [2.784, 3.491]*	2.552 [2.307, 2.811]*	1.226 [1.047, 1.416]*
Fear		0.718 [0.647, 0.795]*	1.274 [1.173, 1.377]*	0.563 [0.492, 0.639]*
Disgust		2.326 [2.083, 2.581]*	2.128 [1.917, 2.347]*	1.094 [0.937, 1.254]
Anger		1.732 [1.554, 1.919]*	1.693 [1.538, 1.856]*	1.023 [0.884, 1.173]

Distributions are summarized using the median and 95% HPDI. Asterisk Represents contrasts where the null value (i.e., 0 for deltas or 1 for ratios) is not contained in the 95% HPDI.

#### Mask effect

**FIGURE 3:**
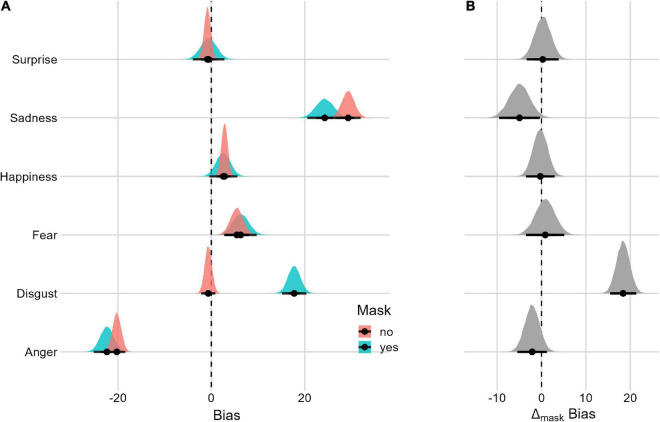
Posterior distributions and 95% HPDIs of the *bias* Mask effect **(A)** *Bias* posterior distributions as a function of the Mask condition. **(B)** Posterior distributions of the Mask Δ contrast (Mask_yes_–Mask_no_).

Figure 3 summarizes each posterior distribution and the Mask effect. Facial expressions of sadness, disgust, fear, anger, and happiness have a *bias* different from 0. For disgust, the *bias* is only present when the face is presented with a facial mask. In terms of the Mask effect, facial expressions of sadness and disgust are associated with different *bias* values. With disgusted faces, in particular, the presence of the mask clearly increases the response *bias.* Despite being smaller, the Mask effect for sad faces is reversed where the presence of the mask reduces the response *bias*. We did not find a Mask effect for a surprise.

#### Mask and facial-expression intensity interaction

**FIGURE 4:**
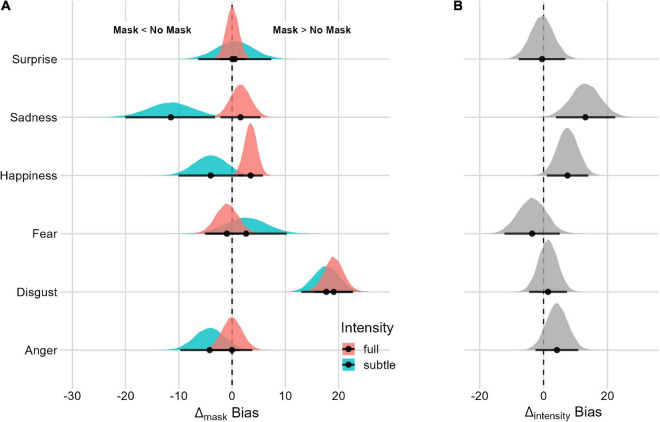
Posterior distributions and 95% HPDIs of the bias Facial Expression Intensity Effect. **(A)** Posterior distributions of Mask Δ as a function of the Facial Expression Intensity condition. **(B)** Posterior distribution of the Facial Expression Intensity Δ contrast (Mask Δ_full_–Mask Δ_subtle_).

We assessed the Mask effect for the subtle and full *Facial-Expression Intensity* (see Figure 4 and Table 2). The model with the three-way interaction (mask, emotion and Facial-Expression Intensity, LOO = −40,133.1, SE = 196.2, p_model_ = 0.723) is 2.6 times more likely than the model without the three-way interaction (LOO = −40,150.5, SE = 195.6, p_model_ = 0.277). The Mask effect differs in relation to subtle and full facial expressions only for facial expressions of sadness and happiness. More specifically, for facial expressions of happiness at full intensity, the bias is greater with the mask. For subtle facial expressions of sadness, the effect is reversed, with greater bias in the condition without the Mask. We did not find a difference in the Mask effect between subtle and full facial expressions when considering faces with a facial expression of surprise.

### Uncertainty

The first model also predicts the *uncertainty* with Mask, Emotion, and Facial-Expression Intensity as predictors. Posterior distribution summaries for the Mask effect and the interaction between Mask and Facial-Expression Intensity are presented respectively in Tables 1, 2.

#### Mask effect

**FIGURE 5:**
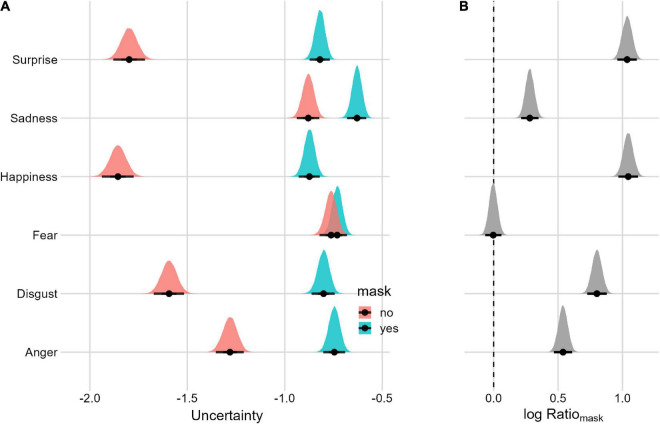
Posterior distributions and 95% HPDIs of the uncertainty Mask effect **(A)** *uncertainty* posterior distributions as a function of the Mask condition. **(B)** Posterior distribution of the Mask Ratio (Mask_yes_/Mask_no_). Values are plotted on the logarithm scale for better visualization (the null value is 0).

For *uncertainty* (Figure 5 and Table 1) we followed the same approach as above. Overall, the *uncertainty* is lower for the condition without the mask. There is evidence of the Mask effect for each emotion except fear.

#### Mask and facial-expression intensity interaction

**FIGURE 6:**
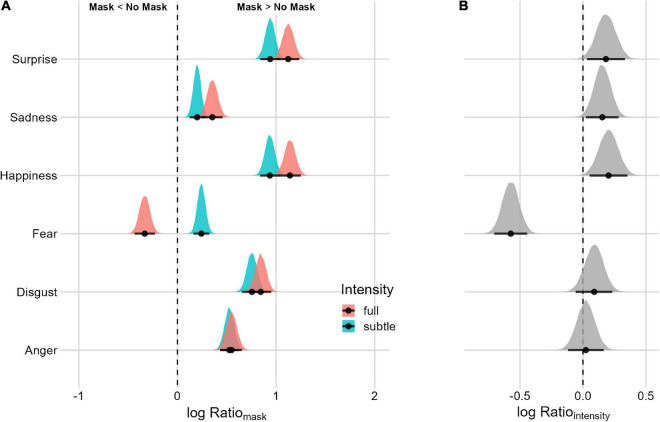
Posterior distributions and 95% HPDIs of the uncertainty Facial Expression Intensity Effect **(A)** uncertainty posterior distributions as a function of the Facial Expression Intensity condition. **(B)** Posterior distribution of the Facial Expression Intensity Ratios (Mask Ratio_full_/Mask Ratio_subtle_). Values are plotted on the logarithm scale for better visualization (the null value is 0).

Assessing the Mask effect for subtle and full facial expressions (Figure 6 and Table 2), there is evidence of a difference in *uncertainty* ratios for facial expressions of surprise, sadness, fear, and happiness. For fearful faces, the Mask effect is reversed between subtle and full facial expressions. When the intensity is subtle, there is more uncertainty in the masked condition, whereas, for full-intensity, expression generates more uncertainty without the mask. For surprise, sadness, and happiness, the Mask effect is present for both full and subtle facial expressions. Despite a smaller effect, when considering the difference between full and subtle intensity, the Mask effect is lower with subtle facial expressions.

### Perceived intensity

The second model predicts *perceived intensity* with Mask, Emotion, and Facial-expression Intensity as predictors. Posterior distribution summaries for the Mask effect and the interaction between Mask and Facial-Expression Intensity are presented respectively in Tables 3, 4.

**TABLE 3:** Posterior distribution summaries for the *perceived intensity* Mask effect as a function of the displayed Emotion.

Emotion	Mask_*yes*_	Mask_*no*_	Contrast
Surprise	137.154 [132.304, 142.094]*	180.893 [175.907, 185.793]*	−43.754 [−46.906, −40.646]*
Sadness	128.319 [123.379, 133.189]*	147.595 [142.71, 152.556]*	−19.311 [−22.343, −16.169]*
Happiness	135.455 [130.544, 140.384]*	173.538 [168.605, 178.442]*	−38.105 [−41.213, −35.021]*
Fear	134.076 [129.218, 139.008]*	155.974 [151.158, 160.997]*	−21.886 [−25.008, −18.867]*
Disgust	166.133 [161.263, 171.101]*	197.035 [192.09, 201.917]*	−30.913 [−34.022, −27.856]*
Anger	153.572 [148.598, 158.344]*	163.772 [158.74, 168.569]*	−10.197 [−13.341, −7.129]*

Distributions are summarized using the median and the 95% HPDI. Asterisks represent contrast where the null value (i.e., 0) is not contained in the 95% HPDI.

**TABLE 4:** Posterior distribution summaries for the perceived intensity Mask effect as a function of the displayed Emotion.

Emotion	Δ *Mask*_full_	Δ *Mask*_subtle_	Contrast
Surprise	−37.287 [−41.586, −32.75]*	−50.215 [−54.675, −45.955]*	12.933 [6.755, 19.033]*
Sadness	−18.173 [−22.324, −13.6]*	−20.443 [−24.766, −16.027]*	2.307 [−3.999, 8.406]
Happiness	−31.522 [−35.959, −27.267]*	−44.677 [−49.076, −40.385]*	13.141 [6.863, 19.188]*
Fear	−10.753 [−15.119, −6.488]*	−33.026 [−37.348, −28.639]*	22.297 [16.116, 28.5]*
Disgust	−22.591 [−26.971, −18.188]*	−39.231 [−43.574, −34.859]*	16.665 [10.384, 22.798]*
Anger	−2.822 [−6.99, 1.658]	−17.58 [−21.988, −13.206]*	14.727 [8.319, 20.722]*

Distributions are summarized using the median and the 95% HPDI. Asterisks represent contrast where the null value (i.e., 0) is not contained in the 95% HPDI.

#### Mask effect

**FIGURE 7:**
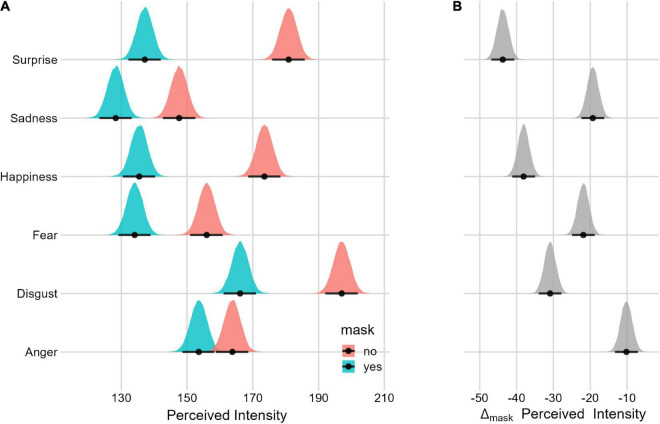
Posterior distributions and 95% HPDIs of the *perceived intensity* Mask effect **(A)** Perceived intensity posterior distributions as a function of the Mask condition. **(B)** Posterior distribution of the Mask Δ (Mask_yes_–Mask_no_).

The *perceived intensity* is generally lower when the mask is present. Figure 7 and Table 3 report the *perceived intensity* in each condition and the Mask effect. There is evidence of the Mask effect for each displayed emotion.

#### Mask and facial-expression intensity interaction

**FIGURE 8:**
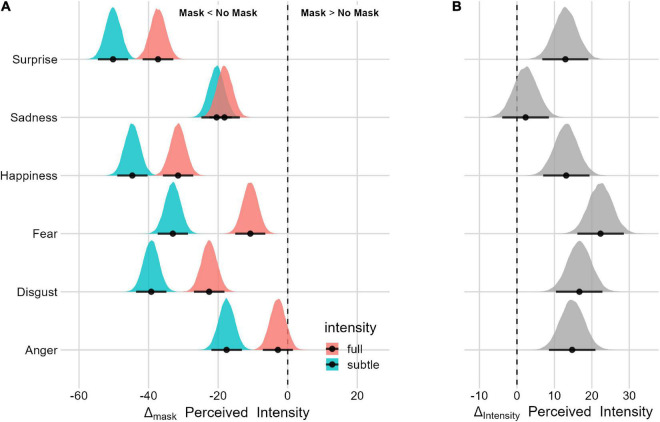
Posterior distributions and 95% HPDI of the perceived intensity Facial Expression Intensity Effect **(A)** Perceived intensity posterior distributions as a function of the Facial Expression Intensity condition. **(B)** Posterior distribution of the Facial Expression Intensity Δ (Mask Δ_full_–Mask Δ_subtle_).

To assess the effect of the facial-expression intensity, we first compared the model with and without the three-way interaction (mask, emotion, and facial-expression intensity). The model with the three-way interaction (LOO = −169,115.6, SE = 127.3, p_model_ = 0.753) is 3 times more likely than the model without the three-way interaction (LOO = −169,121.6, SE = 127.4, p_model_ = 0.247). With the exception of sadness, the Mask effect is greater for subtle facial expressions for each displayed emotion (Figure 8 and Table 4).

### Toronto Alexithymia Scale and Autism Quotient

The average scores of TAS and AQ in our sample were respectively 14.9 (SD = 6.62, IQR = 8) and 52.1 (SD = 8.53, IQR = 12).

We centered TAS and AQ scores and set sum contrasts on the Mask predictor for better interpretability of model parameters (Schad et al., 2020). The TAS has no effect on the response *bias* (β = −0.0002, SE = 0.0004, 95% HPDI = [−0.001, 0.001], *log*BF_01_ = 6.93). Furthermore, there is no interaction between TAS and the presence of the mask (β = −0.0003, SE = 0.0007, 95% HPDI = [−0.0017, 0.001], *log*BF_01_ = 6.47).

There is also no evidence of a TAS effect on the *uncertainty* parameter either for the TAS main effect (β = 0.001, SE = 0.002, 95% HPDI = [−0.003, 0.006], *log*BF_01_ = 5.21) or for the interaction between TAS and Mask (β = 0.001, SE = 0.002, 95% HPDI = [−0.003, 0.004], *log*BF_01_ = 5.53).

When considering only the subtle facial expressions, there is no relationship between *bias* and TAS scores (β = 0.001, SE = 0.001, 95% HPDI = [−0.001, 0.002], *log*BF_01_ = 6.753) and no interaction between TAS and the presence of the mask (β = −0.001, SE = 0.001, 95% HPDI = [−0.004, 0.001], *log*BF_01_ = 6.038). For the *uncertainty*, we found no main effect of TAS (β = 0.002, SE = 0.003, 95% HPDI = [−0.004, 0.008], *log*BF_01_ = 5.592), and no interaction between TAS and the presence of the mask (β = −0.003, SE = 0.003, 95% HPDI = [−0.009, 0.004], *log*BF_01_ = 5.356).

Similarly, we found no evidence either for the relationship between AQ scores and response *bias* (β = −0.001, SE = 0.001, 95% HPDI = [0.002, 00004], *log*BF_01_ = 6.06) or for the interaction between AQ and Mask (β = −0.0001, SE = 0.001, 95% HPDI = [−0.002, 0.002], *log*BF_01_ = 6.30). Similarly, concerning *uncertainty*, we found no evidence for the AQ main effect (β = −0.0006, SE = 0.003 95% HPDI = [−0.006, 0.005], *log*BF_01_ = 5.16) or for the interaction between AQ and Mask (β = −0.0021, SE = 0.0024, 95% HPDI = [−0.007, 0.003], *log*BF_01_ = 5).

When considering subtle facial expressions, we found no relationship between AQ scores and response *bias* (β = −0.001, SE = 0.001, 95% HPDI = [−0.003, 0.001], logBF01 = 5.704), and no interaction between AQ scores and the presence of the Mask (β = −0.001, SE = 0.002, 95% HPDI = [−0.004, 0.003], *log*BF_01_ = 5.524). For the *uncertainty*, we found no AQ main effect (β = −0.002, SE = 0.004, 95% HPDI = [−0.01, 0.006], *log*BF_01_ = 4.767), and no interaction between AQ and the presence of the Mask (β = 0, SE = 0.004, 95% HPDI = [−0.008, 0.009], *log*BF_01_ = 4.768).

In relation to the *perceived intensity*, we found no evidence of a main effect of TAS scores (β = −0.24, SE = 0.27, 95% HPDI = [−0.757, 0.296], *log*BF_01_ = 2.54) or of an interaction between TAS and Mask (β = −0.007, SE = 0.093, 95% HPDI = [−0.188, 0.176], *log*BF_01_ = 3.94). When considering only the subtle facial expressions, we did not found a TAS effect (β = −0.304, SE = 0.330, 95% HPDI = [−0.967, 0.318], *log*BF_01_ = 2.325) or the interaction between TAS and Mask (β = −0.097, SE = 0.122, 95% HPDI = [−0.342, 0.133], *log*BF_01_ = 3.40).

We found the same scenario for the AQ scores. There was no evidence of a main effect of AQ scores (β = −0.331, SE = 0.349, 95% HPDI = [−1.01, 0.355], *log*BF_01_ = 2.3) or of an interaction between AQ and Mask (β = −0.146, SE = 0.119, 95% HPDI = [−0.38, 0.086], *log*BF_01_ = 3). When considering only subtle facial expressions, again, we did not find a AQ effect (β = −0.145, SE = 0.421, 95% HPDI = [−0.947, 0.69], *log*BF_01_ = 2.40) or the interaction between AQ and Mask (β = −0.147, SE = 0.156, 95% HPDI = [−0.444, 0.167], *log*BF_01_ = 3.02).

Overall, when considering just the subtle facial expressions, we still found evidence for the absence of effect on *perceived intensity, uncertainty*, and *bias*.

## Discussion

This study aims to provide a comprehensive description of the types of errors committed when trying to recognize full and subtle basic facial expression expressed by faces covered by a mask. To this end, we asked the participants to respond using a Geneva Emotion Wheel, intending to define their performance according to three indices that we believe could provide a more precise picture of the impact of masks on facial-expression recognition: (1) uncertainty, i.e., the tendency to provide responses associated with different emotional labels without this necessarily being associated with a systematic misattribution of emotional expressions; (2) bias, i.e., the systematic error of confusing one emotion with others; and (3) perceived intensity.

Notably and not surprisingly, the uncertainty in the participants’ responses (i.e., the amount of spread in responses) increases for *all facial expressions* (except for *fear)* when faces are covered by a mask. However, when considering the intensity of the expression, *subtle expressions of fear* are also associated with an increase in uncertainty. Interestingly, in the study by Carbon (2020), the author measured participants’ confidence for each assessment of the facial-expression recognition task on a scale from 1 (very unconfident) to 7 (very confident) and found a large-sized effect for all the expressions tested. Our results dovetail nicely with these previous findings by providing an index that is not based on the subjectively felt confidence about one’s assessment but, rather, an objective measure of such confidence (i.e., uncertainty). In brief, we believe that our results align perfectly with these previous findings, while using a more fine-grained performance index (i.e., uncertainty) based on the GEW complex space.

Regarding response bias, our results indicate a tendency to systematically confuse the expression of *disgust* with other emotions (especially anger: see Figures 2, 3) when a face is masked. This result is not surprising considering that the prototypical expression of disgust is characterized by the curling of the nose (in terms of AUs, this corresponds to AU9) and the lifting of the upper lip (AU10). Therefore, diagnostic information is incomplete, or almost completely missing when faces are covered by a mask (in particular, with the use of the N95 mask, which tends to cover not only the mouth region but also the nose completely). This result appears to align with previous studies (Carbon, 2020; Carbon and Serrano, 2021; Ziccardi et al., 2021; Carbon et al., 2022; Kim et al., 2022; Tsantani et al., 2022).

The results relating to the expression of *sadness*, on the other hand, may appear surprising. These, albeit marginally, indicate a reversed bias when faces are covered by a mask (i.e., fostering the correct recognition of sadness expressed by masked faces: see Figure 3). Indeed, previous studies (e.g., Carbon, 2020; Carbon and Serrano, 2021; Carbon et al., 2022; Kim et al., 2022) have reported a worsening in the recognition of sadness expressed by a masked face. However, considering the AUs available when a face is masked, the one prototypically associated with sadness is AU1 (i.e., a raising and approaching of the eyebrows). Since no other facial expression of a primary emotion has these characteristics, it seems legitimate to conclude that the presence of a mask may allow a person to focus on the most diagnostic and available information for recognizing sadness. It should be noted that other studies did not observe a decrease in the recognition of sadness when the face was covered by the mask (see Noyes et al., 2021). Indeed, when considering the intensity of the expressions, subtle sad faces are associated with an increase in response bias when faces are covered by masks.

We did not observe an increase/decrease in response bias for the remaining emotions (i.e., *surprise*, *fear, anger*, and *happiness)* as a function of the mask. As far as surprise, fear, and anger are concerned, this result seems in line with the observation that most distinctive information remains available despite the mask covering the face. The eye region is the most important of all three of these expressions. On the other hand, the result relating to happiness is unexpected since the mask hides the mouth and the contraction of the zygomatic muscle that is markedly associated with this expression. However, the mask leaves another diagnostic element visible, namely, that relating to the eyes and the contraction of the orbicular muscle in its external part, which may be sufficient for the correct detection of the expression. It should be noted that, when also considering the expression intensity as a function of the impact of the mask, full-intensity expressions of happiness are associated with a slight response bias, particularly toward the categories of interest and surprise.

Finally, *all the expressions* (especially surprise and happiness) were perceived as less intense when covered by a mask. This finding aligns nicely with previous studies (Pazhoohi et al., 2021; Sheldon et al., 2021; Kastendieck et al., 2022; Langbehn et al., 2022; Ramachandra and Longacre, 2022; Tsantani et al., 2022). Interestingly and with the exception of sadness, we have found that this effect is even more pronounced when the expressions are subtle.

To summarize the main results, the presence of a mask makes the recognition of all primary emotions more demanding (as supported by the “uncertainty” index) regardless of their level of intensity. The one exception is intense fear, which is not subject to this increment of uncertainty as a function of the face mask manipulation. Overall, these findings indicate that face masks reduce diagnostic information for recognizing facial expressions. Moreover, when they do not induce systematic errors of emotion misattribution, they increase uncertainty in observers regarding which emotion the other person is feeling/communicating. The observation that this uncertainty also increases for subtle fear when the face is masked supports our hypothesis that the processing of subtle expressions may be more markedly affected by face masks.

On the other hand, an increase in systematic misattribution errors (i.e., “bias”) when the faces are covered by a mask (vs. uncovered) seems to be minimal and mainly concerns the expression of disgust, which is primarily confused with anger. This is so, regardless of the degree of the expression’s intensity. To a lesser extent, the full expression of happiness is also confused with interest and surprise, and the subtle expressions of sadness are misinterpreted as fear and anger. Thus, these misattribution errors seem to concern only those expressions that are distinctively conveyed by the (covered) lower portion of the face (i.e., disgust and happiness).

Finally, all the emotions were perceived as less intense, especially when subtle.

Overall, these findings suggest that face masks affect the recognition of emotions differently according to the availability of the diagnostic information distributed over the face. Thus, the emotions mainly conveyed by the lower portion of the face are more likely to be subjected to misattribution errors, while all emotions, especially those conveyed by the upper portion of the face, are associated with a general increase in uncertainty.

Although the present study did not directly investigate the neural basis of expression perception in conditions of mask covering (mainly because of the protracted closure of the department’s electroencephalography and neuroimaging laboratories due to the pandemic), we believe some considerations may be helpful to future studies interested in investigating such neural underpinnings.

In particular, we expect that uncertainty - as we have measured it in the present work - could have a neural counterpart, since there is evidence that uncertainty of participants’ responses is linked with variability in neural responses (Festa et al., 2021).

What kind of neural responses could present this kind of variability? To provide an answer to this question, we need to consider neural markers of face processing and the most accredited neural model for processing faces and facial expressions. Three principal posterior brain areas are involved in the visual processing of faces (Haxby and Gobbini, 2011; Duchaine and Yovel, 2015; see also Dalrymple et al., 2011), the fusiform face area (FFA), the occipital face area (OFA), and the posterior superior temporal sulcus (pSTS). FFA is considered the main neural substrate of configural-holistic face processing (e.g., Mazard et al., 2006; Schiltz and Rossion, 2006), while pSTS is sensitive to changing features, such as facial expressions (Haxby and Gobbini, 2011; Duchaine and Yovel, 2015). It is reasonable to assume that the mask has an impact on the holistic-configural processing of faces (and therefore on the activation of FFA) and that in the conditions in which the face is covered by the mask, the processing of facial expressions of emotion may be devolved mainly to the OFA and pSTS. However, when a face is covered by a mask, OFA and pSTS have a reduced amount of information available compared to when the face is fully visible. Although at the moment, this is only speculation, it is plausible that the (reduced and partial) diagnostic/distinctive information for emotion recognition when a mask is worn is associated with greater variability of neural responses in these regions, hence, resulting in a decrease in perceptual sensitivity and an increase in the uncertainty of participants’ responses (see Festa et al., 2021). At the electrophysiological level, even the most well-known marker of face processing originating from these posterior regions, namely, the N170 event-related potential, could reflect this increase in uncertainty in the form of a latency delay or a greater latency variability.

When the mask covers those features that strongly characterize an expression of emotion, any features still available in the upper portion of the face can induce misattribution errors if they are a diagnostic of other primary emotions. This would seem to be precisely what was observed for the expression of disgust. The only additional secondary feature available when the face is covered is the tension of the inferior eyelid (i.e., AU7), a diagnostic feature of anger. In this case, these misattribution errors would not be primarily associated with increased variability in neuronal responses in the OFA and pSTS but, rather, with the “correct” analysis of the available relevant—but misleading—information.

Finally, the most recent sensorimotor simulation model considers that the involvement of a distributed emotion system during the processing of expressions of emotion supports their recognition (Wood et al., 2016). This system is recruited either directly by the exposure to expressions of emotions or indirectly by the sensorimotor system. The observation that a mask reduces the experienced intensity of emotions suggests that this emotion system is recruited to a lesser extent when the expressions are covered by a mask than when they are completely visible. It is interesting to note that this result, in some respects, mimics the performance of patients with ventromedial prefrontal cortex lesions whose judgment about the intensity of facial expressions does not correspond to the actual intensity of such expressions, unlike patients with other (non-critical) prefrontal lesions and healthy control subjects (Heberlein et al., 2008).

We also expected to observe a relationship between alexithymic and autistic traits assessed by means of the 20-item TAS (Bagby et al., 1994) and the AQ (Baron-Cohen et al., 2001) with the three indices (uncertainty, bias, and perceived intensity). To our knowledge, our study is the first to use a large sample to explore the relationship between alexithymic and autistic traits and performance in emotion recognition as a function of the face mask. Surprisingly, we did not find any evidence of such relations. These results are even more surprising if one considers that the ability to read emotions from the eye region is particularly compromised in several neuropsychiatric disorders, including autism spectrum disorder (e.g., Baron-Cohen et al., 2001). Furthermore, there is evidence that performance on the Reading the Mind in the Eyes Test (RMET) is impaired in alexithymic individuals (Oakley et al., 2016; Rødgaard et al., 2019). These results might suggest that the recognition performance relating to a face’s eye area (as in RMET) is not entirely comparable to the recognition performance relating to facial expressions covered by the mask. However, caution is necessary to accept these conclusions definitively. Indeed, the low scores’ variability in the questionnaires to measure alexithymic and autistic traits could limit the possibility of observing a relationship between performance and these traits. It is also important to underline that our sample comprises healthy subjects, and in a few cases, we have observed scores above the clinical cut-offs.

Regarding the present study’s limitations, we note that due to the safety regulations introduced to prevent exposure to the SARS-COV-2 virus, most studies on this topic (including ours) have, to date, been conducted online. While this has allowed larger numbers of participants to collaborate in the different studies, it is also true that it has allowed only limited control over the experiments’ settings. Another limitation concerns the nature of the stimuli presented. More often than not, the facial expressions portraying the targeted effect displayed it in a stereotypical manner and with exaggerated intensity, using photographs of actors who have received instructions regarding which muscles to contract to achieve the desired expression. In everyday life, however, the facial expressions people are confronted with may be different, more sophisticated, less obvious, and therefore, harder to categorize. We tried to overcome these limitations, at least in part, by manipulating the expressions’ intensity (full vs. subtle), demonstrating that the processing of subtle expressions is even more compromised by face masks. It must also be stressed that many studies had higher numbers of female than male participants, making it difficult to carry out gender comparisons. Our study, too, is subject to this limitation, as most of the participants were women. It is not clear how this gender unbalance could have influenced the results of our and previous studies. We imagine two alternative scenarios, both based on experimental evidence: We see two possible and opposite scenarios: (1) Since it is known that women are more expressive than men (e.g., Kring and Gordon, 1998), more accurate in processing emotional expressions (e.g., Hoffmann et al., 2010) and more empathetic (e.g., Singer and Lamm, 2009), this gender unbalance could lead to an underestimation of the impact of face masks on the ability to recognize facial expressions; and (2) On the other side, it has been proposed that women are better at recognizing emotional expressions because they use a more embodied route (see, e.g., Stel and van Knippenberg, 2008), which could be strongly affected by the covering of the lower part of the face. This evidence might lead to opposite conclusions that samples made almost entirely from women can produce an overestimation of the impact of face masks on the ability to recognize facial expressions.

Another possible limitation is based on the knowledge about neural models of face processing: When processing expressions of emotion, the activation would further propagate throughout the dorsal pathway to more anterior regions (anterior superior temporal sulcus, aSTS, and inferior frontal gyrus; Duchaine and Yovel, 2015). In addition, sensorimotor and embodied simulation models assign a central role to the frontal operculum, the ventromedial prefrontal cortex, the supplementary motor area, and the emotion system (Gallese, 2005; Wood et al., 2016; Gallese and Sinigaglia, 2018). It is known that these brain areas are more strongly recruited by dynamic (rather than static) facial expressions (Duchaine and Yovel, 2015; Pitcher and Ungerleider, 2021). From this point of view, it is possible that, in ecological conditions, the misattribution errors and participants’ uncertainty observed in our study may be attenuated by the additional information conveyed by the movement of the facial muscles involved in the facial expression.

To conclude, we also believe that the present study has some merits. First of all, the introduction of the GEW to collect the participants’ responses probably reduced the possibility of ceiling effects. It also allowed us to identify more clearly the misattribution errors that may involve secondary emotions (for example happiness being confused with interest as well as a surprise). Furthermore, thanks to the use of the GEW’s complex space, we were able to compute an objective index of uncertainty in the participants’ responses: one which seems to correspond to the results regarding response confidence found in previous studies (Carbon, 2020). In general, using this tool to gather participants’ answers allowed us to obtain rich information about the perception-space of facial expressions of emotion in terms of bias, uncertainty, and perceived intensity. This though a single click for each expression presented. These indices permitted us to clarify that the emotions conveyed mainly by the lower portion of the face (covered by the mask) are more likely to be associated with response bias. All emotions, including those characterized by elements peculiar to the upper portion of the face (not covered), are subject to increased response uncertainty. Furthermore, when covered by a mask, all emotions are perceived as less intense, and this is particularly so when they are subtly expressed.

## Data availability statement

The datasets presented in this study can be found in online repositories. The names of the repository/repositories and accession number(s) can be found below: Open Science Framework; https://osf.io/e2kcw/.

## Ethics statement

The studies involving human participants were reviewed and approved by Comitato Etico Della Ricerca Psicologica—Area 17—Università degli Studi di Padova. The patients/participants provided their written informed consent to participate in this study.

## Author contributions

PS developed the study concept. AV programmed the experiment and prepared the stimuli. AV and CR gathered the data. FG performed the data analysis, while PS and FG interpreted the data. All authors contributed to the study design, drafted the manuscript, and approved the final version for submission.

## Footnotes

1 “The Facial Action Coding System (FACS; Ekman and Friesen, 1978) is a comprehensive, anatomically based system for describing all visually discernible facial movement. It breaks down facial expressions into individual components of muscle movement, called Action Units (AUs).” (From https://www.paulekman.com/facial-action-coding-system/).

2
https://www.kdef.se/

3
https://github.com/alyssaq/face_morpher

4
https://github.com/aqeelanwar/MaskTheFace

5
http://www.gorilla.sc/

6
https://osf.io/e2kcw/
