# Supplementary material for: Mapping the perception-space of facial expressions in the era of face masks
Source: Front Psychol. 2022 Sep 13;13:956832. doi: 10.3389/fpsyg.2022.956832 (PMC9514388; doi:10.3389/fpsyg.2022.956832)
Supplement: Supplementary file 1 [file Data_Sheet_1.PDF]

Supplementary Materials

Contents

1 General approach **3**
1.1 Facial expression category . . . 3
1.2 Perceived Intensity . . . 3
1.3 Statistical models . . . 4
1.3.1 `brms` . . . 4
1.4 Raw data . . . 5

2 Fitted Models **7**
2.1 Bias/Uncertainty . . . 11
2.1.1 fit_ri_aq_mask . . . 11
2.1.2 fit_ri_aq_mask_subtle . . . 12
2.1.3 fit_ri_int . . . 13
2.1.4 fit_ri_neu . . . 15
2.1.5 fit_ri_no3int . . . 16
2.1.6 fit_ri_tas_mask . . . 18
2.1.7 fit_ri_tas_mask_subtle . . . 19
2.2 Perceived intensity . . . 20
2.2.1 fit_ri_aq_mask . . . 20
2.2.2 fit_ri_aq_mask_subtle . . . 21
2.2.3 fit_ri_int . . . 22
2.2.4 fit_ri_neu . . . 24
2.2.5 fit_ri_no3int . . . 25
2.2.6 fit_ri_tas_mask . . . 26
2.2.7 fit_ri_tas_mask_subtle . . . 27

3 Priors Sensitivity **28**
3.1 Bias/Uncertainty . . . 28
3.1.1 fit_ri_aq_mask_subtle_un . . . 28
3.1.2 fit_ri_aq_mask_un . . . 30
3.1.3 fit_ri_int_un . . . 31
3.1.4 fit_ri_tas_mask_subtle_un . . . 33
3.1.5 fit_ri_tas_mask_un . . . 34
3.2 Perceived Intensity . . . 35
3.2.1 fit_ri_aq_mask_flat . . . 35
3.2.2 fit_ri_aq_mask_subtle_flat . . . 36
3.2.3 fit_ri_int_flat . . . 37
3.2.4 fit_ri_tas_mask_flat . . . 39
3.2.5 fit_ri_tas_mask_subtle_flat . . . 40

4 Suggestions for meta-analysis **41**
4.1 Bias . . . 41
4.2 Uncertainty . . . 41
4.3 Perceived Intensity . . . 41

References **42**

1 General approach

The Geneva Emotion Wheel [GEW; Scherer (2005)] allows having an intuitive and informative way to collect participants' responses in a facial expression perception task. Specifically, in a single measurement is possible to have information about the facial expression *category* (i.e., the response angle around the circle) and *intensity* (i.e., the distance from the center).

1.1 Facial expression category

In order to measure the response angle for each trial we transformed Cartesian coordinates ((x_i, y_i)) into polar coordinates ((r_i, θ_i)) as in Equation (1).

$$\theta_{ij} = tan^{-1}(\frac{y_{ij}}{x_{ij}}) \tag{1}$$

In this way we have the *pressed angle* for each trial. Given that each emotion has an absolute location on the GEW, we calculated a *position-free* index of performance computing the difference between the *pressed angle* and the *ideal angle* (i.e., the GEW location of the presented emotion).

Then we calculated the *ideal* angle for each presented emotion, in the middle of each wheel circle. To obtain a measure comparable between emotion, we calculated the angular difference between the *ideal* and the pressed angle using the Equation (2)

$$Bias = ((ideal - pressed) + 180) \mod 360 - 180 \tag{2}$$

This new measure (*bias*) has several advantages. Despite each emotion have a different location within the wheel, each response is now expressed in a position-free metric. The *bias* is centered on 0 if there is no response tendency away from the *ideal* value. Otherwise, a systematic shift would move the circular mean away from 0, clockwise (positive values) or anticlockwise (negative values). Other than the circular mean, also the spread on the circle (i.e., *uncertainty*) is an important performance measure. The *bias* and the *uncertainty* are can be considered independent measures.

Given the periodicity of circular data, we cannot use standard statistical modeling tools (Cremers, Mulder, and Klugkist 2018; Cremers and Klugkist 2018). There are different ways to model circular data (see Cremers, Mulder, and Klugkist 2018 for an overview). We decided to use a generalized linear mixed-effect model using the *von Mises* likelihood function. The *von Mises* distribution is an alternative to the Gaussian distribution for circular data, bounded in the range $[-\pi, \pi]$. The two parameters of the von Mises distribution, μ and k[1] representing our *bias* and *uncertainty* parameters. To facilitate the interpretation of models' parameters, we transformed k into the circular variance using Equation (3).

$$\sigma^2 = 1 - \frac{I_1(k)}{I_0(k)} \tag{3}$$

The circular variance ranges between 0 (no *uncertainty*) to 1 (maximum *uncertainty*). The transformation is computed using the modified Bessel function $I_i(k)$ of order i (Evans, Hastings, and Peacock 2011).

1.2 Perceived Intensity

The emotion *intensity* is expressed as the difference from the center of the GEW. Values close or far from the center represent respectively neutral and high facial expression intensity. We calculated the *intensity* for each trial as the *euclidean distance* between the *center* and the *pressed location.* Given that the GEW has been centered (i.e., the center has coordinates $x = 0, y = 0$), the distance from the center is calculated as Equation (4).

[1] In fact, k is a concentration parameter that can be conceptually considered as the inverse of the standard deviation. When the concentration is 0 the distribution is *uniform*

$$I_{ij} = \sqrt{x^2 + y^2} \tag{4}$$

1.3 Statistical models

For the response angle (i.e., *bias* and *uncertainty*) we decided to use a *scale-location* mixed-effect model (Bürkner 2018; Rigby and Stasinopoulos 2005). Under this framework, all parameters of a distribution can be predicted. In particular, we are predicting the *circular mean* (i.e., *bias*) and the *concentration* (i.e., *uncertainty*) Von Mises parameters as a function of Mask (with or without), Intensity (full and subtle) and Emotion (anger, happiness, disgust, fear, surprise and sadness). For the perceived intensity, we used a regular general linear mixed-effect model.

We estimated both models under a Bayesian framework the R software (R Core Team 2021) using the Brms package (Bürkner 2017) based on the STAN probabilistic programming language (Carpenter et al. 2017). The Bayesian statistics consist in combining information from prior knowledge (i.e. *priors*) and the data (i.e., *likelihood*) to obtain the *posterior* distribution (Kruschke and Liddell 2018).

In terms of contrast coding, for categorical predictors, we used `sum contrasts` using the `contr.sum()` function. We used sum-contrasts with `0.5, -0.5` only for TAS and AQ models for interpreting directly the model parameters. We also mean-centered numeric predictors (TAS and AQ scores).

1.3.1 `brms`

We fitted our models using the `brms` package. According to different models the `brm` setup could be different in terms of `backend`, number of `iterations` and `chains` and the parallelization approach. The general approach for `bias/uncertainty` models is the following:

```
# the scale-location specification
form <- bf(theta_cen ~ ... + (1|id),
           kappa ~ ... + (1|id))

brm(formula, # model formula
    data = data,
    prior = priors,
    family = von_mises(link = "tan_half", link_kappa = "log"),
    chains = 15,
    cores = 15,
    iter = 4000,
    backend = "cmdstanr", # or the standard backend, depending on how to setup the parallelization
    sample_prior = "yes",
    save_pars = save_pars(all = TRUE),
    seed = 2022)
```

For the `perceived intensity`

```
brm(int ~ ... + (1|id),
    data = data,
    prior = priors,
    family = gaussian(),
    chains = 15,
    cores = 15,
    iter = 4000,
    backend = "cmdstanr", # or the standard backend, depending on how to setup the parallelization
    save_pars = save_pars(all = TRUE),
    sample_prior = "yes",
    seed = 2022)
```

When fitting models with uninformative or flat priors, we used a different chains/iteration approach to improve model fitting (especially for the Von Mises model). In particular we used the *within-chains* parallelization (https://cran.r-project.org/web/packages/brms/vignettes/brms_threading.html) for `bias/uncertainty` models:

```
# the scale-location specification
form <- bf(theta_cen ~ ... + (1|id),
           kappa ~ ... + (1|id))

brm(form,
    data = data,
    family = von_mises(link = "tan_half", link_kappa = "log"),
    chains = 4,
    prior = priors, # uninformative or flat
    cores = 4,
    iter = 10000,
    sample_prior = "yes",
    backend = "cmdstanr",
    threads = threading(6),  # within-chains parallellization
    save_pars = save_pars(all = TRUE),
    seed = seed)
```

For the `perceived intensity` models we use the same approach as the main models given the simpler fitting process.

1.4 Raw data

The figure S2 represents all participants' responses for each experimental condition, directly plotted on the GEW. The figure S1 represents the GEW legend and the responses to the neutral condition.

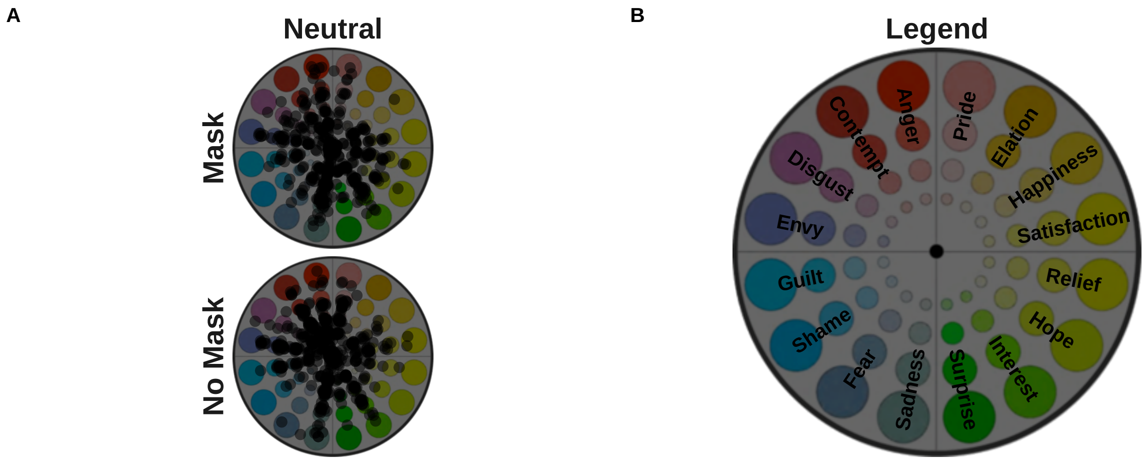

Figure S1: GEW legend (B) and responses to neutral facial expressions as a function of the Mask condition (A)

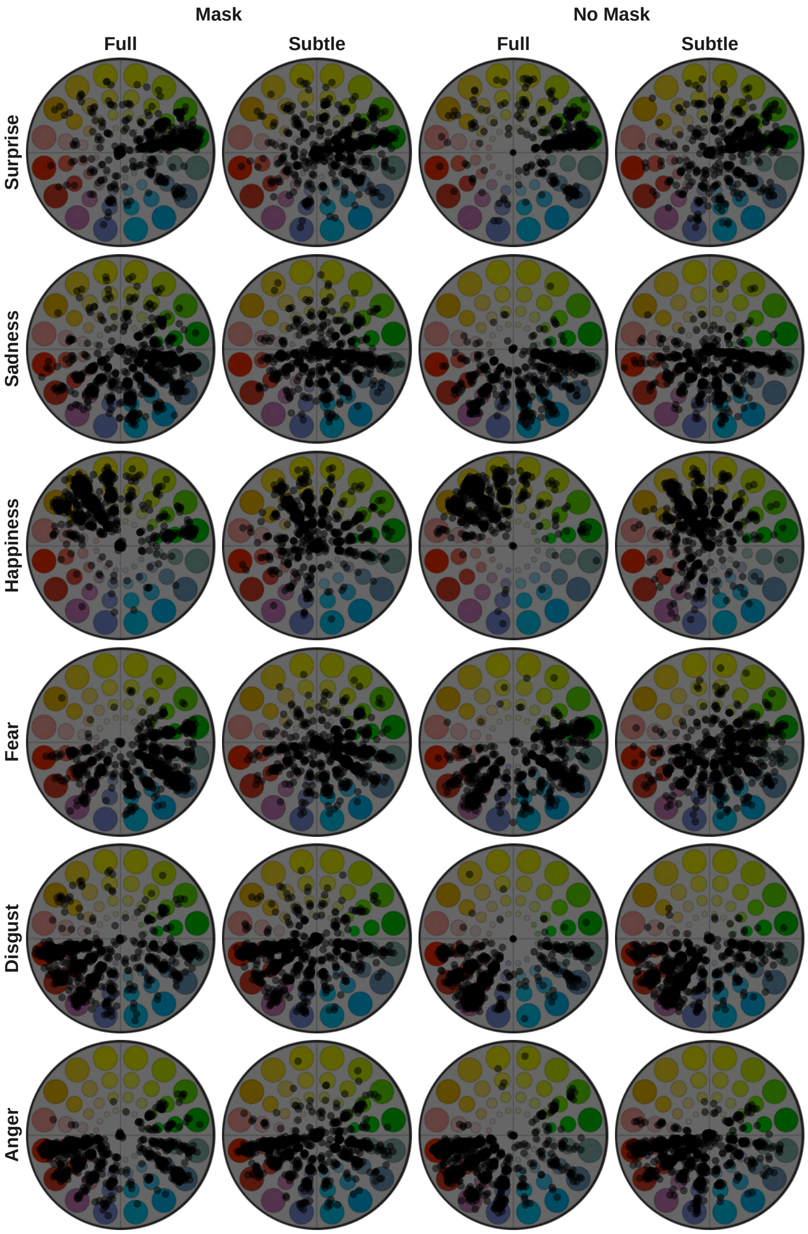

Figure S2: GEW responses as a function of displayed Emotion, Mask and Facial Expression Intensity.

2 Fitted Models

Table S1 depicts all fitted models with main parameters. To read the table:

- `fit_ri_int`: the random-intercept three-way interaction model for *bias/uncertainty* and *perceived intensity*
- `fit_ri_no3int`: the random-intercept model without the three-way interaction *bias/uncertainty* and *perceived intensity*
- `fit_ri_aq_mask`: the random-intercept model with the Autism-Spectrum Quotient test (AQ) for *bias/uncertainty* and *perceived intensity* (Mask effect)
- `fit_ri_tas_mask`: the random-intercept model with the Toronto Alexithymia Scale (TAS) *bias/uncertainty* and *perceived intensity* (Mask effect)
- `fit_ri_tas_subtle`: the random-intercept model with the Toronto Alexithymia Scale (TAS) *bias/uncertainty* and *perceived intensity* (Mask effect only for subtle facial expressions)
- `fit_ri_tas_subtle`: the random-intercept model with the Autism-Spectrum Quotient test (AQ) for *bias/uncertainty* and *perceived intensity* (Mask effect only for subtle facial expressions)
- `fit_*un/flat`: models with completely uninformative or flat priors

Table S1: Table with model formulas, names and fitting parameters

<table>
<tr><th>model</th><th>name</th><th>chains</th><th>iter</th><th>warmup</th><th>samples</th></tr>
<tr><td>diff_theta ~ 0 + Intercept + mask_e * aq + (1 | id)
kappa ~ 0 + Intercept + mask_e * aq + (1 | id)</td><td>fit_ri_aq_mask_subtle_un</td><td>4</td><td>10,000</td><td>5,000</td><td>20,000</td></tr>
<tr><td>diff_theta ~ 0 + Intercept + mask_e * aq + (1 | id)
kappa ~ 0 + Intercept + mask_e * aq + (1 | id)</td><td>fit_ri_aq_mask_subtle</td><td>15</td><td>4,000</td><td>2,000</td><td>30,000</td></tr>
<tr><td>diff_theta ~ 0 + Intercept + mask_e * aq + (1 | id)
kappa ~ 0 + Intercept + mask_e * aq + (1 | id)</td><td>fit_ri_aq_mask_un</td><td>4</td><td>10,000</td><td>5,000</td><td>20,000</td></tr>
<tr><td>diff_theta ~ 0 + Intercept + mask_e * aq + (1 | id)
kappa ~ 0 + Intercept + mask_e * aq + (1 | id)</td><td>fit_ri_aq_mask</td><td>15</td><td>4,000</td><td>2,000</td><td>30,000</td></tr>
<tr><td>diff_theta ~ emotion * mask * intensity + (1 | id)
kappa ~ emotion * mask * intensity + (1 | id)</td><td>fit_ri_int_un</td><td>4</td><td>10,000</td><td>5,000</td><td>20,000</td></tr>
<tr><td>diff_theta ~ emotion * mask * intensity + (1 | id)
kappa ~ emotion * mask * intensity + (1 | id)</td><td>fit_ri_int</td><td>15</td><td>4,000</td><td>2,000</td><td>30,000</td></tr>
<tr><td>theta_cen ~ 0 + Intercept + mask_e + (1 | id)
kappa ~ 0 + Intercept + mask_e + (1 | id)</td><td>fit_ri_neu</td><td>15</td><td>4,000</td><td>2,000</td><td>30,000</td></tr>
<tr><td>diff_theta ~ emotion + mask + intensity + emotion:mask +
emotion:intensity +
mask:intensity + (1 | id)
kappa ~ emotion + mask + intensity + emotion:mask +
emotion:intensity +
mask:intensity + (1 | id)</td><td>fit_ri_no3int</td><td>15</td><td>4,000</td><td>2,000</td><td>30,000</td></tr>
<tr><td>diff_theta ~ 0 + Intercept + mask_e * tas + (1 | id)
kappa ~ 0 + Intercept + mask_e * tas + (1 | id)</td><td>fit_ri_tas_mask_subtle_un</td><td>4</td><td>10,000</td><td>5,000</td><td>20,000</td></tr>
<tr><td>diff_theta ~ 0 + Intercept + mask_e * tas + (1 | id)
kappa ~ 0 + Intercept + mask_e * tas + (1 | id)</td><td>fit_ri_tas_mask_subtle</td><td>4</td><td>10,000</td><td>5,000</td><td>20,000</td></tr>
<tr><td>diff_theta ~ 0 + Intercept + mask_e * tas + (1 | id)
kappa ~ 0 + Intercept + mask_e * tas + (1 | id)</td><td>fit_ri_tas_mask_un</td><td>4</td><td>10,000</td><td>5,000</td><td>20,000</td></tr>
<tr><td>diff_theta ~ 0 + Intercept + mask_e * tas + (1 | id)
kappa ~ 0 + Intercept + mask_e * tas + (1 | id)</td><td>fit_ri_tas_mask</td><td>15</td><td>4,000</td><td>2,000</td><td>30,000</td></tr>
<tr><td>int ~ 0 + Intercept + mask_e * aq + (1 | id)</td><td>fit_ri_aq_mask_flat</td><td>15</td><td>4,000</td><td>2,000</td><td>30,000</td></tr>
<tr><td>int ~ 0 + Intercept + mask_e * aq + (1 | id)</td><td>fit_ri_aq_mask_subtle_flat</td><td>15</td><td>4,000</td><td>2,000</td><td>30,000</td></tr>
</table>

model	name	chains	iter	warmup	samples
int ~ 0 + Intercept + mask_e * aq + (1 | id)	fit_ri_aq_mask_subtle	15	4,000	2,000	30,000
int ~ 0 + Intercept + mask_e * aq + (1 | id)	fit_ri_aq_mask	15	4,000	2,000	30,000
int ~ 0 + Intercept + emotion * mask * intensity + (1 | id)	fit_ri_int_flat	15	4,000	2,000	30,000
int ~ 0 + Intercept + emotion * mask * intensity + (1 | id)	fit_ri_int	15	4,000	2,000	30,000
int ~ 0 + Intercept + mask + (1 | id)	fit_ri_neu	15	4,000	2,000	30,000
int ~ 0 + Intercept + emotion + mask + intensity + emotion:mask + emotion:intensity + mask:intensity + (1 | id)	fit_ri_no3int	15	4,000	2,000	30,000
int ~ 0 + Intercept + mask_e * tas + (1 | id)	fit_ri_tas_mask_flat	15	4,000	2,000	30,000
int ~ 0 + Intercept + mask_e * tas + (1 | id)	fit_ri_tas_mask_subtle_flat	15	4,000	2,000	30,000
int ~ 0 + Intercept + mask_e * tas + (1 | id)	fit_ri_tas_mask_subtle	15	4,000	2,000	30,000
int ~ 0 + Intercept + mask_e * tas + (1 | id)	fit_ri_tas_mask	15	4,000	2,000	30,000

In the next section we presented all fitted models using the same approach:

- the model name (the same name as the R object)
- prior distributions for each parameter
- model output

For the prior tables:

- `prior`: is the prior distribution with parameters. All parameters without a proper prior (i.e., different from a flat prior) are not reported in the table.
- `class`: is the type of parameter (`b` is for β and `sd` for a standard deviation parameter e.g., by-subject intercept or residual σ)
- `coef`: is the specific model parameters. If a prior is defined only for a *class*, then all parameters of that class will have the same prior
- `dpar`: is for *distributional parameters*. In the case of the von Mises model refers to k coefficients

For the model tables:

- `param`: is the model parameter name
- `estimate`: is the mean of the posterior distribution
- `Est.Error`: is the standard error of the posterior distribution
- `95% CI`: is the 95% credible interval
- `Rhat`: is the Gelman and Rubin (Gelman and Rubin 1992) convergence index. When is below 1.1 the parameters has converged.
- `Bulk/Tail Effective Sample Size`: can be considered as the amount of information used for estimating a parameter. In general higher is better (see https://mc-stan.org/docs/2_18/reference-manual/effective-sample-size-section.html). Is calculated from the number of iterations and chains of the models.

2.1 Bias/Uncertainty

2.1.1 fit_ri_aq_mask

2.1.1.1 Priors

Table S2:

prior	class	coef	dpar
normal(0, 1)	b	aq	
normal(0, 5)	b	Intercept	
normal(0, 5)	b	mask_e1	
normal(0, 1)	b	mask_e1:aq	
normal(0, 1)	b	aq	kappa
normal(0, 5)	b	Intercept	kappa
normal(0, 5)	b	mask_e1	kappa
normal(0, 1)	b	mask_e1:aq	kappa
student_t(3, 0, 2.5)	sd		
student_t(3, 0, 2.5)	sd		kappa

2.1.1.2 Model

Table S3:

param	median	se	lower	upper	Rhat	Bulk_ESS	Tail_ESS
aq	-0.00070	0.00056	-0.00181	0.00039	1.00006	34,470.55778	26,671.54697
Intercept	0.02250	0.00363	0.01533	0.02946	0.99992	37,393.30208	24,795.23276
kappa_aq	-0.00058	0.00291	-0.00643	0.00497	1.00084	11,700.25669	17,939.41680
kappa_Intercept	0.44895	0.01957	0.41078	0.48745	1.00069	10,579.91240	15,800.56698
kappa_mask_e1	0.49709	0.01575	0.46691	0.52876	1.00057	54,067.32872	21,159.17014
kappa_mask_e1:aq	-0.00205	0.00237	-0.00673	0.00260	1.00076	46,612.10273	20,810.26913
mask_e1	-0.02705	0.00602	-0.03867	-0.01522	1.00019	54,126.34790	23,119.24072
mask_e1:aq	-0.00013	0.00093	-0.00195	0.00169	1.00017	33,051.44573	20,007.58417
sd(Intercept)	0.02260	0.00480	0.01327	0.03202	1.00146	9,409.61710	11,607.95569
sd(kappa_Intercept)	0.19957	0.01558	0.17022	0.23084	1.00080	10,308.50947	16,087.89041

2.1.2 fit_ri_aq_mask_subtle

2.1.2.1 Priors

Table S4:

prior	class	coef	dpar
normal(0, 0.5)	b	aq	
normal(0, 2)	b	Intercept	
normal(0, 2)	b	mask_e1	
normal(0, 0.5)	b	mask_e1:aq	
normal(0, 0.5)	b	aq	kappa
normal(0, 2)	b	Intercept	kappa
normal(0, 2)	b	mask_e1	kappa
normal(0, 0.5)	b	mask_e1:aq	kappa
student_t(3, 0, 2.5)	sd		
student_t(3, 0, 2.5)	sd		kappa

2.1.2.2 Model

Table S5:

param	median	se	lower	upper	Rhat	Bulk_ESS	Tail_ESS
aq	-0.00104	0.00098	-0.00292	0.00088	1.00054	38,196.81231	26,318.45495
Intercept	0.03198	0.00610	0.01982	0.04366	1.00043	29,706.42249	22,790.62141
kappa_aq	-0.00175	0.00395	-0.00957	0.00595	1.00113	10,085.15169	15,069.15421
kappa_Intercept	0.08478	0.02635	0.03231	0.13597	1.00207	9,140.76703	14,467.38122
kappa_mask_e1	0.69697	0.02776	0.64057	0.74941	1.00046	34,889.36440	22,662.70167
kappa_mask_e1:aq	0.00045	0.00421	-0.00765	0.00868	1.00075	44,468.72941	22,177.97040
mask_e1	-0.01705	0.01185	-0.04030	0.00578	1.00051	31,667.33943	23,185.26848
mask_e1:aq	-0.00061	0.00189	-0.00435	0.00305	1.00080	52,230.16278	20,474.92991
sd(Intercept)	0.01184	0.00857	0.00000	0.02835	1.00200	7,208.66817	10,749.24749
sd(kappa_Intercept)	0.24749	0.02216	0.20499	0.29124	1.00152	9,133.92208	16,174.14485

2.1.3 fit_ri_int

2.1.3.1 Priors

Table S6:

prior	class	coef	dpar
normal(0, 5)	b		
normal(0, 5)	b		kappa
student_t(3, 0, 2.5)	Intercept		
normal(5.0, 0.8)	Intercept		kappa
student_t(3, 0, 2.5)	sd		
student_t(3, 0, 2.5)	sd		kappa

2.1.3.2 Model

Table S7:

param	median	se	lower	upper	Rhat	Bulk_ESS	Tail_ESS
emotion1	-0.22085	0.00724	-0.23487	-0.20664	1.00032	30,735.28980	24,944.70656
emotion1:intensity1	0.03690	0.00720	0.02291	0.05100	1.00028	30,906.23100	25,149.15927
emotion1:mask1	0.01812	0.00723	0.00398	0.03229	1.00009	31,030.20968	23,943.20511
emotion1:mask1:intensity1	-0.00102	0.00720	-0.01521	0.01302	1.00016	31,224.24675	24,856.62223
emotion2	0.04261	0.00649	0.03009	0.05539	1.00010	32,447.36394	25,165.23496
emotion2:intensity1	0.00284	0.00646	-0.00959	0.01574	1.00005	32,106.25240	25,470.41521
emotion2:mask1	-0.07245	0.00647	-0.08514	-0.05992	1.00016	31,978.63676	25,404.94644
emotion2:mask1:intensity1	0.00560	0.00654	-0.00744	0.01815	1.00051	32,368.35253	24,951.87120
emotion3	0.01873	0.00851	0.00200	0.03526	1.00016	27,725.41199	24,713.97740
emotion3:intensity1	0.00532	0.00859	-0.01186	0.02183	1.00011	27,869.38302	24,237.32723
emotion3:mask1	0.00482	0.00865	-0.01212	0.02202	1.00031	27,607.55290	23,640.64445
emotion3:mask1:intensity1	0.01635	0.00868	-0.00031	0.03359	1.00011	27,747.58626	24,278.91250
emotion4	-0.00908	0.00685	-0.02249	0.00421	1.00037	19,976.20777	22,924.98143
emotion4:intensity1	0.00211	0.00681	-0.01122	0.01538	1.00018	20,508.78806	23,731.32894
emotion4:mask1	0.00969	0.00687	-0.00372	0.02297	1.00019	20,327.18021	23,642.86262
emotion4:mask1:intensity1	-0.00789	0.00684	-0.02168	0.00525	1.00028	20,511.97001	23,599.41091
emotion5	0.20738	0.00976	0.18815	0.22651	1.00040	22,814.52684	22,874.10413
emotion5:intensity1	-0.05956	0.00969	-0.07847	-0.04052	1.00033	23,809.69165	23,496.40597
emotion5:mask1	0.03260	0.00967	0.01353	0.05141	1.00018	23,049.96262	23,911.69982

param	median	se	lower	upper	Rhat	Bulk__ESS	Tail__ESS
emotion5:mask1:intensity1	-0.02267	0.00973	-0.04143	-0.00328	1.00031	22,909.25069	23,679.02604
intensity1	-0.01463	0.00350	-0.02152	-0.00778	1.00020	31,286.91333	25,490.71399
Intercept	0.03259	0.00393	0.02482	0.04019	1.00005	25,530.97457	24,028.24958
kappa__emotion1	-0.06890	0.01840	-0.10658	-0.03448	1.00038	40,204.81488	23,421.65594
kappa__emotion1:intensity1	-0.19129	0.01837	-0.22680	-0.15487	1.00067	42,684.72589	23,438.35321
kappa__emotion1:mask1	-0.04303	0.01834	-0.07829	-0.00692	1.00060	43,036.58609	24,537.74442
kappa__emotion1:mask1:intensity1	0.01850	0.01826	-0.01715	0.05422	1.00084	41,038.19824	23,894.26255
kappa__emotion2	0.10296	0.01799	0.06860	0.13881	1.00079	45,505.51030	24,595.70588
kappa__emotion2:intensity1	-0.24953	0.01774	-0.28488	-0.21498	1.00025	45,625.06928	23,945.57109
kappa__emotion2:mask1	0.05702	0.01803	0.02260	0.09294	1.00062	44,006.45377	23,972.34198
kappa__emotion2:mask1:intensity1	0.04661	0.01766	0.01223	0.08131	1.00063	43,263.53400	23,722.02577
kappa__emotion3	-0.35866	0.02087	-0.40038	-0.31885	1.00020	36,432.17354	24,215.93988
kappa__emotion3:intensity1	-0.06833	0.02052	-0.10976	-0.02970	0.99994	37,051.84128	24,330.78846
kappa__emotion3:mask1	-0.30189	0.02059	-0.34334	-0.26257	1.00035	35,138.81287	24,534.82978
kappa__emotion3:mask1:intensity1	-0.13876	0.02060	-0.17967	-0.09851	1.00003	37,543.77476	23,693.18583
kappa__emotion4	0.37359	0.01932	0.33545	0.41116	1.00000	34,557.61635	24,197.81084
kappa__emotion4:intensity1	0.20805	0.01893	0.17159	0.24559	1.00017	36,801.48734	25,183.42775
kappa__emotion4:mask1	0.20714	0.01924	0.16930	0.24452	1.00009	38,597.47485	23,828.93160
kappa__emotion4:mask1:intensity1	0.03435	0.01912	-0.00276	0.07231	1.00073	38,335.38768	23,923.88370
kappa__emotion5	-0.36122	0.02181	-0.40439	-0.31898	1.00048	31,095.31525	23,657.65207
kappa__emotion5:intensity1	0.05253	0.02151	0.01086	0.09467	1.00016	33,216.49740	23,321.93137
kappa__emotion5:mask1	-0.14408	0.02161	-0.18599	-0.10132	1.00015	33,113.15853	23,790.85544
kappa__emotion5:mask1:intensity1	0.03204	0.02153	-0.01015	0.07478	1.00077	32,871.19584	23,375.38191
kappa__intensity1	0.41878	0.00884	0.40165	0.43627	1.00008	46,839.76466	24,182.64803
kappa__Intercept	0.56793	0.02450	0.52037	0.61640	1.00279	4,832.75265	10,156.83382
kappa__mask1	0.32903	0.00886	0.31089	0.34556	1.00036	46,787.05993	24,955.01875
kappa__mask1:intensity1	-0.05211	0.00875	-0.06911	-0.03486	1.00015	48,550.40092	23,847.89418
mask1	-0.00846	0.00348	-0.01524	-0.00164	1.00056	30,933.96526	25,042.72497
mask1:intensity1	-0.00852	0.00350	-0.01528	-0.00159	1.00046	29,871.64201	24,443.79403
sd(Intercept)	0.01966	0.00345	0.01301	0.02657	1.00126	12,378.65687	14,414.33770
sd(kappa__Intercept)	0.26047	0.01931	0.22406	0.29940	1.00099	7,640.79115	13,349.70183

2.1.4 fit_ri_neu

2.1.4.1 Priors

Table S8:

prior	class	coef	dpar
uniform(-3.141593, 3.141593)	b		
normal(0, 2)	b		kappa
student_t(3, 0, 2.5)	sd		
student_t(3, 0, 2.5)	sd		kappa

2.1.4.2 Model

Table S9:

param	median	se	lower	upper	Rhat	Bulk_ESS	Tail_ESS
Intercept	-0.53885	0.06858	-0.67584	-0.40812	1.00273	6,520.04931	11,032.75032
kappa_Intercept	-0.59686	0.09336	-0.78654	-0.42319	1.00191	8,762.64159	15,102.70238
kappa_mask_e1	0.29382	0.11227	0.08311	0.52395	1.00007	21,498.74190	22,169.20293
mask_e1	-0.17323	0.06506	-0.30068	-0.04495	1.00024	22,311.67187	22,107.31209
sd(Intercept)	0.51920	0.07648	0.37584	0.67327	1.00348	5,189.28148	10,323.32665
sd(kappa_Intercept)	0.53913	0.09171	0.36611	0.72529	1.00190	6,598.64126	13,111.42743

2.1.5 fit__ri__no3int

2.1.5.1 Priors

Table S10:

prior	class	coef	dpar
normal(0, 2)	b		
normal(0, 2)	b		kappa
student_t(3, 0, 2.5)	Intercept		
normal(5.0, 0.8)	Intercept		kappa
student_t(3, 0, 2.5)	sd		
student_t(3, 0, 2.5)	sd		kappa

2.1.5.2 Model

Table S11:

param	median	se	lower	upper	Rhat	Bulk__ESS	Tail__ESS
emotion1	-0.22078	0.00687	-0.23412	-0.20734	1.00007	47,510.10436	26,013.69501
emotion1:intensity1	0.03671	0.00638	0.02454	0.04953	1.00015	58,235.38944	23,183.93895
emotion1:mask1	0.01870	0.00640	0.00640	0.03138	1.00071	55,668.57518	24,058.40440
emotion2	0.04117	0.00630	0.02877	0.05341	1.00014	45,703.25292	26,700.70693
emotion2:intensity1	0.00607	0.00556	-0.00450	0.01730	1.00048	58,768.58355	24,557.86467
emotion2:mask1	-0.06973	0.00600	-0.08155	-0.05802	1.00025	51,390.67732	24,902.60113
emotion3	0.01508	0.00818	-0.00110	0.03080	1.00015	47,344.43652	24,384.27958
emotion3:intensity1	0.00802	0.00829	-0.00803	0.02447	1.00014	46,926.85334	24,956.77573
emotion3:mask1	0.01425	0.00704	0.00008	0.02757	1.00051	65,565.85812	22,198.81843
emotion4	-0.00439	0.00593	-0.01581	0.00741	1.00058	39,352.46643	25,067.04934
emotion4:intensity1	-0.00346	0.00531	-0.01390	0.00696	1.00020	44,206.74702	25,372.35848
emotion4:mask1	0.00428	0.00481	-0.00521	0.01368	1.00081	50,680.82896	24,073.24112
emotion5	0.21334	0.00942	0.19506	0.23197	1.00023	34,361.61009	25,919.44804
emotion5:intensity1	-0.06682	0.00919	-0.08519	-0.04911	1.00012	36,551.59572	25,672.58994
emotion5:mask1	0.01720	0.00705	0.00358	0.03100	1.00049	53,030.95562	21,882.83128
intensity1	-0.01552	0.00344	-0.02231	-0.00888	1.00022	38,581.38563	26,879.21795
Intercept	0.03318	0.00389	0.02552	0.04080	1.00011	34,986.27417	26,402.12032
kappa__emotion1	-0.06785	0.01828	-0.10374	-0.03223	1.00073	59,458.58829	23,542.68415
kappa__emotion1:intensity1	-0.19150	0.01820	-0.22804	-0.15615	1.00069	64,207.12475	21,997.86408

param	median	se	lower	upper	Rhat	Bulk_ESS	Tail_ESS
kappa_emotion1:mask1	-0.03766	0.01811	-0.07312	-0.00192	1.00038	66,002.13451	22,602.50904
kappa_emotion2	0.10032	0.01790	0.06649	0.13683	1.00040	62,257.03616	23,178.85206
kappa_emotion2:intensity1	-0.24562	0.01790	-0.27988	-0.20954	1.00038	63,801.19447	22,791.39758
kappa_emotion2:mask1	0.06452	0.01791	0.03020	0.10033	1.00073	61,052.42396	23,743.81695
kappa_emotion3	-0.34722	0.02017	-0.38824	-0.30900	1.00055	53,693.06046	25,212.55945
kappa_emotion3:intensity1	-0.07884	0.02009	-0.11822	-0.03975	1.00051	54,501.43796	24,324.55940
kappa_emotion3:mask1	-0.34478	0.01909	-0.38135	-0.30695	1.00018	64,072.27197	21,971.08882
kappa_emotion4	0.36615	0.01896	0.32882	0.40322	1.00036	57,541.87406	23,361.60829
kappa_emotion4:intensity1	0.21260	0.01902	0.17512	0.24955	1.00040	55,574.97033	25,411.23974
kappa_emotion4:mask1	0.21716	0.01910	0.18099	0.25578	1.00065	56,352.92534	24,427.68307
kappa_emotion5	-0.36560	0.02126	-0.40726	-0.32429	1.00061	49,636.54865	25,914.15323
kappa_emotion5:intensity1	0.05727	0.02126	0.01710	0.09968	1.00032	53,120.62710	26,023.29446
kappa_emotion5:mask1	-0.12765	0.01958	-0.16718	-0.09000	1.00030	61,563.77383	22,522.07210
kappa_intensity1	0.42188	0.00891	0.40414	0.43920	1.00008	58,039.43617	24,881.42581
kappa_Intercept	0.56525	0.02492	0.51649	0.61446	1.00187	6,932.36293	12,222.05205
kappa_mask1	0.32605	0.00881	0.30877	0.34311	1.00048	61,063.50977	24,071.37000
kappa_mask1:intensity1	-0.04914	0.00869	-0.06640	-0.03245	1.00033	64,799.39401	25,185.02115
mask1	-0.01000	0.00331	-0.01643	-0.00344	1.00022	47,182.67499	25,191.02213
mask1:intensity1	-0.00720	0.00328	-0.01363	-0.00087	1.00038	42,506.07866	26,265.92500
sd(Intercept)	0.01952	0.00343	0.01267	0.02615	1.00075	12,456.26973	16,368.16676
sd(kappa_Intercept)	0.25944	0.01918	0.22379	0.29834	1.00130	9,293.53132	17,528.15932

2.1.6 fit__ri__tas__mask

2.1.6.1 Priors

Table S12:

prior	class	coef	dpar
normal(0, 7)	b	Intercept	
normal(0, 7)	b	mask__e1	
normal(0, 2)	b	mask__e1:tas	
normal(0, 2)	b	tas	
normal(0, 7)	b	Intercept	kappa
normal(0, 7)	b	mask__e1	kappa
normal(0, 2)	b	mask__e1:tas	kappa
normal(0, 2)	b	tas	kappa
student__t(3, 0, 2.5)	sd		
student__t(3, 0, 2.5)	sd		kappa

2.1.6.2 Model

Table S13:

param	median	se	lower	upper	Rhat	Bulk__ESS	Tail__ESS
Intercept	0.02255	0.00369	0.01513	0.02960	1.00022	37,559.84117	25,925.63343
kappa__Intercept	0.44862	0.01950	0.40986	0.48658	1.00114	8,734.27644	14,220.77221
kappa__mask__e1	0.49717	0.01548	0.46705	0.52787	1.00124	69,488.06242	22,080.34858
kappa__mask__e1:tas	0.00063	0.00184	-0.00299	0.00416	1.00121	50,494.15403	19,311.35625
kappa__tas	0.00138	0.00228	-0.00317	0.00576	1.00130	10,509.53826	16,681.89598
mask__e1	-0.02690	0.00593	-0.03854	-0.01537	1.00114	68,274.83185	21,639.67255
mask__e1:tas	-0.00033	0.00069	-0.00168	0.00104	1.00030	30,058.33889	19,493.29313
tas	-0.00022	0.00043	-0.00109	0.00061	1.00002	33,781.45265	26,294.45526
sd(Intercept)	0.02323	0.00464	0.01428	0.03262	1.00045	11,117.66908	14,160.65426
sd(kappa__Intercept)	0.19939	0.01567	0.17019	0.23110	1.00155	10,830.03016	17,961.96772

2.1.7 fit_ri_tas_mask_subtle

2.1.7.1 Priors

Table S14:

prior	class	coef	dpar
normal(0, 5)	b	Intercept	
normal(0, 5)	b	mask_e1	
normal(0, 1)	b	mask_e1:tas	
normal(0, 1)	b	tas	
normal(0, 5)	b	Intercept	kappa
normal(0, 5)	b	mask_e1	kappa
normal(0, 1)	b	mask_e1:tas	kappa
normal(0, 1)	b	tas	kappa
student_t(3, 0, 2.5)	sd		
student_t(3, 0, 2.5)	sd		kappa

2.1.7.2 Model

Table S15:

param	median	se	lower	upper	Rhat	Bulk_ESS	Tail_ESS
Intercept	0.03187	0.00609	0.01993	0.04378	1.00010	21,262.68042	15,879.66581
kappa_Intercept	0.08542	0.02590	0.03651	0.13858	1.00035	8,662.92080	11,587.86143
kappa_mask_e1	0.69691	0.02793	0.64212	0.75187	1.00052	28,068.76515	14,697.95216
kappa_mask_e1:tas	-0.00292	0.00325	-0.00908	0.00375	1.00007	28,797.50078	15,554.45666
kappa_tas	0.00189	0.00308	-0.00422	0.00780	1.00059	8,688.67841	11,916.91714
mask_e1	-0.01665	0.01189	-0.03938	0.00733	1.00043	23,513.04593	15,417.20951
mask_e1:tas	-0.00147	0.00136	-0.00411	0.00120	1.00017	30,162.08421	14,602.93701
tas	0.00069	0.00071	-0.00075	0.00205	1.00041	24,980.70468	16,906.02488
sd(Intercept)	0.01236	0.00881	0.00000	0.02933	1.00093	5,187.09305	8,264.33409
sd(kappa_Intercept)	0.24642	0.02205	0.20564	0.29201	1.00054	6,767.36908	10,467.16001

2.2 Perceived intensity

2.2.1 fit_ri_aq_mask

2.2.1.1 Priors

Table S16:

prior	class	coef	dpar
student_t(3, 0, 78.1)	sd		
student_t(3, 0, 78.1)	sigma		

2.2.1.2 Model

Table S17:

param	median	se	lower	upper	Rhat	Bulk_ESS	Tail_ESS
aq	-0.33080	0.34974	-1.01022	0.35516	1.00293	2,137.48528	4,601.62671
Intercept	156.36900	2.35099	151.66900	160.88300	1.00800	1,739.79951	3,208.98398
mask_e1	27.35400	0.78208	25.81170	28.87190	1.00062	37,962.28333	21,785.11431
mask_e1:aq	-0.14647	0.11883	-0.38017	0.08569	1.00058	38,139.89850	20,416.30572
sd(Intercept)	25.65950	1.70644	22.50290	29.10810	1.00699	2,599.26047	4,264.65119

2.2.2 fit_ri_aq_mask_subtle

2.2.2.1 Priors

Table S18:

prior	class	coef	dpar
normal(0, 5)	b	aq	
normal(150, 100)	b	Intercept	
normal(0, 50)	b	mask_e1	
normal(0, 5)	b	mask_e1:aq	
student_t(3, 0, 72.6)	sd		
student_t(3, 0, 72.6)	sigma		

2.2.2.2 Model

Table S19:

param	median	se	lower	upper	Rhat	Bulk_ESS	Tail_ESS
aq	-0.14484	0.42064	-0.94749	0.68971	1.00331	2,331.25183	3,991.90022
Intercept	121.24500	2.75593	115.86200	126.67500	1.00869	1,974.11939	3,790.55303
mask_e1	34.17445	1.02796	32.18110	36.17230	1.00032	39,470.67483	21,971.32260
mask_e1:aq	-0.14661	0.15597	-0.44368	0.16731	1.00133	37,139.21688	22,105.13145
sd(Intercept)	31.08145	2.10112	27.20070	35.33610	1.00438	3,048.28879	4,748.53848

2.2.3 fit_ri_int

2.2.3.1 Priors

Table S20:

prior	class	coef	dpar
student_t(3, 0, 78.1)	sd		
student_t(3, 0, 78.1)	sigma		

2.2.3.2 Model

Table S21:

param	median	se	lower	upper	Rhat	Bulk_ESS	Tail_ESS
emotion1	2.54529	0.71593	1.14247	3.94736	1.00049	41,987.03568	22,493.99203
emotion1:intensity1	-5.07667	0.72007	-6.45963	-3.64553	1.00044	42,718.83792	22,575.97752
emotion1:mask1	-8.57880	0.72701	-10.03310	-7.17673	1.00028	47,496.95729	22,900.70566
emotion1:mask1:intensity1	-0.26581	0.72366	-1.61670	1.19070	1.00038	42,679.05223	22,588.26013
emotion2	25.46390	0.72790	24.02680	26.86670	1.00029	43,561.20092	20,758.85648
emotion2:intensity1	-9.39143	0.71800	-10.76450	-7.94710	1.00084	42,778.15059	22,879.57226
emotion2:mask1	1.77797	0.71823	0.30590	3.11976	1.00019	43,667.67337	22,828.72061
emotion2:mask1:intensity1	-0.74968	0.72366	-2.12668	0.69295	1.00053	42,356.27447	22,591.71846
emotion3	-11.09680	0.71935	-12.51490	-9.68895	1.00033	44,053.54897	23,211.99958
emotion3:intensity1	2.21339	0.72465	0.77576	3.60650	1.00014	43,910.42651	21,820.45148
emotion3:mask1	-2.73160	0.71200	-4.11033	-1.34332	1.00026	44,127.20120	23,472.11014
emotion3:mask1:intensity1	-2.15486	0.71838	-3.55349	-0.76253	1.00066	42,279.79896	23,060.71319
emotion4	-1.63321	0.71847	-3.00589	-0.17602	1.00015	44,353.24542	22,061.55022
emotion4:intensity1	8.05619	0.72333	6.64505	9.45965	1.00053	46,101.45040	23,340.06617
emotion4:mask1	5.37099	0.71412	4.00750	6.81193	1.00077	44,837.18793	23,078.90515
emotion4:mask1:intensity1	0.13645	0.71904	-1.29189	1.53183	1.00024	44,931.49664	23,239.01975
emotion5	-18.17580	0.72391	-19.57570	-16.75030	1.00048	44,298.41420	22,500.11365
emotion5:intensity1	0.36721	0.71867	-1.04749	1.76941	1.00088	44,806.46966	22,655.34994
emotion5:mask1	-4.02686	0.72195	-5.44788	-2.61122	1.00029	43,413.79301	22,532.26390
emotion5:mask1:intensity1	2.84715	0.72321	1.45015	4.29105	1.00017	46,054.79602	22,130.11162
intensity1	34.92925	0.32799	34.28640	35.55520	1.00017	53,748.23158	22,162.74403
Intercept	156.12000	2.27925	151.69800	160.55100	1.01169	1,213.89864	2,255.13190
mask1	13.68060	0.32031	13.04080	14.29110	1.00082	56,569.44902	21,859.72920

param	median	se	lower	upper	Rhat	Bulk__ESS	Tail__ESS
mask1:intensity1	-3.41976	0.32295	-4.05492	-2.78889	1.00065	51,871.12710	21,809.84659
sd(Intercept)	25.76450	1.68941	22.64660	29.13390	1.00502	2,371.53985	4,703.46438

2.2.4 fit_ri_neu

2.2.4.1 Priors

Table S22:

prior	class	coef	dpar
normal(0, 50)	b		
normal(150, 100)	b	Intercept	
student_t(3, 0, 7.7)	sd		
student_t(3, 0, 7.7)	sigma		

2.2.4.2 Model

Table S23:

param	median	se	lower	upper	Rhat	Bulk_ESS	Tail_ESS
Intercept	52.95975	3.77819	45.56160	60.44870	1.00328	3,879.06768	8,070.22197
maskyes	-5.56753	2.17690	-9.84766	-1.31022	1.00030	50,186.62614	22,654.66591
sd(Intercept)	38.84835	2.70519	33.88720	44.41130	1.00196	6,161.72137	11,565.56113

2.2.5 fit__ri__no3int

2.2.5.1 Priors

Table S24:

prior	class	coef	dpar
normal(0, 50)	b		
normal(150, 100)	b	Intercept	
student_t(3, 0, 78.1)	sd		
student_t(3, 0, 78.1)	sigma		

2.2.5.2 Model

Table S25:

param	median	se	lower	upper	Rhat	Bulk_ESS	Tail_ESS
emotion1	2.54188	0.71702	1.12845	3.93561	1.00013	48,203.54515	22,691.10803
emotion1:intensity1	-5.07714	0.71537	-6.43047	-3.64033	1.00098	45,809.71787	22,995.93459
emotion1:mask1	-8.58023	0.72137	-10.02160	-7.21794	1.00073	45,189.72337	22,778.79016
emotion2	25.46165	0.72147	24.08200	26.89440	1.00065	42,558.82456	22,305.98581
emotion2:intensity1	-9.38955	0.71917	-10.80870	-7.98139	1.00066	47,041.29366	23,850.26093
emotion2:mask1	1.78351	0.72120	0.36916	3.19063	1.00064	46,087.63256	22,699.23120
emotion3	-11.10270	0.72345	-12.52440	-9.71088	1.00068	44,505.65930	22,002.43773
emotion3:intensity1	2.20767	0.72119	0.73480	3.56951	1.00094	43,683.55404	21,355.19373
emotion3:mask1	-2.73895	0.72219	-4.11268	-1.29020	1.00058	44,575.07658	23,438.79057
emotion4	-1.63552	0.73220	-3.10638	-0.22699	1.00071	45,365.20768	22,850.03999
emotion4:intensity1	8.04563	0.72359	6.64920	9.48108	1.00085	45,136.61476	22,042.84358
emotion4:mask1	5.37190	0.71742	3.97343	6.78193	1.00059	44,111.24088	22,617.17807
emotion5	-18.16210	0.72119	-19.60030	-16.75850	1.00023	46,525.82382	22,411.45620
emotion5:intensity1	0.36802	0.72202	-1.01452	1.80441	1.00042	47,086.34779	22,995.16187
emotion5:mask1	-4.02367	0.71966	-5.43975	-2.61682	1.00098	47,769.98509	22,220.04975
intensity1	34.92740	0.32201	34.29920	35.56090	1.00071	55,076.23006	21,741.37759
Intercept	156.12800	2.33228	151.71500	160.71900	1.01529	1,263.87071	2,965.63322
mask1	13.67570	0.32264	13.03460	14.30560	1.00036	56,252.38995	20,822.03080
mask1:intensity1	-3.41723	0.32518	-4.05721	-2.79488	1.00040	57,802.66029	23,350.14126
sd(Intercept)	25.73600	1.65412	22.62150	29.03160	1.00551	2,346.78020	5,156.66720

2.2.6 fit_ri_tas_mask

2.2.6.1 Priors

Table S26:

prior	class	coef	dpar
student_t(3, 0, 78.1)	sd		
student_t(3, 0, 78.1)	sigma		

2.2.6.2 Model

Table S27:

param	median	se	lower	upper	Rhat	Bulk_ESS	Tail_ESS
Intercept	156.15500	2.31594	151.61300	160.72100	1.00732	1,811.81295	3,692.24722
mask_e1	27.36025	0.77748	25.81190	28.84820	1.00073	45,055.31688	22,214.03531
mask_e1:tas	-0.00677	0.09290	-0.18837	0.17649	1.00104	41,547.51643	21,178.75258
tas	-0.23521	0.26953	-0.75713	0.29640	1.00490	2,318.41246	4,755.98810
sd(Intercept)	25.68600	1.66770	22.54610	29.04000	1.00750	2,806.64043	4,966.52619

2.2.7 fit__ri__tas__mask__subtle

2.2.7.1 Priors

Table S28:

prior	class	coef	dpar
normal(150, 100)	b	Intercept	
normal(0, 50)	b	mask__e1	
normal(0, 5)	b	mask__e1:tas	
normal(0, 5)	b	tas	
student__t(3, 0, 72.6)	sd		
student__t(3, 0, 72.6)	sigma		

2.2.7.2 Model

Table S29:

param	median	se	lower	upper	Rhat	Bulk__ESS	Tail__ESS
Intercept	121.35700	2.78264	115.76300	126.75400	1.00897	1,781.80130	3,347.30251
mask__e1	34.18415	1.03938	32.11880	36.16700	1.00087	37,923.82422	21,368.93120
mask__e1:tas	-0.09720	0.12196	-0.34191	0.13303	1.00042	36,329.42099	21,917.34140
tas	-0.30401	0.32973	-0.96658	0.31779	1.00942	1,821.81939	4,242.57144
sd(Intercept)	30.98540	2.06994	27.11130	35.18880	1.00552	3,079.77105	4,095.38507

3 Priors Sensitivity

In this section, using the same approach as before, we presented the same set of models fitted using very uninformative or flat priors. The rationale is to assess the impact of our main priors specification on parameter values. Comparing the values of the parameters with models in Section 2, there is almost no effect of our priors specification. Some models (especially TAS and AQ models) could have different priors on similar parameters depending on model complexity and convergence issues.

3.1 Bias/Uncertainty

3.1.1 fit_ri_aq_mask_subtle_un

3.1.1.1 Priors

Table S30:

prior	class	coef	dpar
normal(0, 1)	b	aq	
normal(0, 5)	b	Intercept	
normal(0, 5)	b	mask_e1	
normal(0, 1)	b	mask_e1:aq	
normal(0, 1)	b	aq	kappa
normal(0, 5)	b	Intercept	kappa
normal(0, 5)	b	mask_e1	kappa
normal(0, 1)	b	mask_e1:aq	kappa
student_t(3, 0, 2.5)	sd		
student_t(3, 0, 2.5)	sd		kappa

3.1.1.2 Model

Table S31:

param	median	se	lower	upper	Rhat	Bulk_ESS	Tail_ESS
aq	-0.00107	0.00098	-0.00298	0.00088	1.00017	24,677.74623	16,670.85364
Intercept	0.03195	0.00607	0.02008	0.04377	1.00010	19,666.92050	15,458.57143
kappa_aq	-0.00174	0.00392	-0.00950	0.00568	1.00051	6,967.93344	11,757.85305
kappa_Intercept	0.08526	0.02619	0.03282	0.13560	1.00039	6,642.19462	9,911.94488
kappa_mask_e1	0.69684	0.02772	0.64333	0.75128	1.00050	26,190.56083	14,893.89842
kappa_mask_e1:aq	0.00047	0.00426	-0.00759	0.00900	1.00030	25,861.26475	14,960.66929
mask_e1	-0.01700	0.01183	-0.03962	0.00658	1.00031	20,984.00386	15,125.11499
mask_e1:aq	-0.00059	0.00189	-0.00440	0.00296	0.99995	28,181.85518	14,489.18514
sd(Intercept)	0.01207	0.00855	0.00000	0.02855	1.00087	5,081.56120	7,730.11965

param	median	se	lower	upper	Rhat	Bulk_ESS	Tail_ESS
sd(kappa_Intercept)	0.24661	0.02179	0.20663	0.29141	1.00067	6,513.58823	11,906.30580

3.1.2 fit_ri_aq_mask_un

3.1.2.1 Priors

Table S32:

prior	class	coef	dpar
normal(0, 0.5)	b	aq	
normal(0, 2)	b	Intercept	
normal(0, 2)	b	mask_e1	
normal(0, 0.5)	b	mask_e1:aq	
normal(0, 0.5)	b	aq	kappa
normal(0, 2)	b	Intercept	kappa
normal(0, 2)	b	mask_e1	kappa
normal(0, 0.5)	b	mask_e1:aq	kappa
student_t(3, 0, 2.5)	sd		
student_t(3, 0, 2.5)	sd		kappa

3.1.2.2 Model

Table S33:

param	median	se	lower	upper	Rhat	Bulk_ESS	Tail_ESS
aq	-0.00070	0.00056	-0.00175	0.00041	1.00003	19,730.14993	17,005.20099
Intercept	0.02249	0.00360	0.01539	0.02949	0.99995	17,690.00679	16,015.33546
kappa_aq	-0.00062	0.00293	-0.00627	0.00527	1.00082	4,441.84565	7,969.58475
kappa_Intercept	0.44829	0.01922	0.41098	0.48634	1.00036	3,860.65076	6,871.39091
kappa_mask_e1	0.49725	0.01577	0.46577	0.52758	1.00012	32,180.38592	14,809.61228
kappa_mask_e1:aq	-0.00206	0.00236	-0.00669	0.00252	1.00020	37,381.74752	15,128.99389
mask_e1	-0.02705	0.00592	-0.03839	-0.01522	1.00015	27,377.19157	14,581.75665
mask_e1:aq	-0.00015	0.00092	-0.00198	0.00160	1.00019	37,932.44484	14,475.87990
sd(Intercept)	0.02269	0.00484	0.01314	0.03221	1.00011	5,769.04661	5,842.45239
sd(kappa_Intercept)	0.19922	0.01560	0.16958	0.23002	1.00025	5,976.02318	10,066.80723

3.1.3 fit_ri_int_un

3.1.3.1 Priors

Table S34:

prior	class	coef	dpar
normal(0, 2)	b		
normal(0, 2)	b		kappa
student_t(3, 0, 2.5)	Intercept		
normal(5.0, 0.8)	Intercept		kappa
student_t(3, 0, 2.5)	sd		
student_t(3, 0, 2.5)	sd		kappa

3.1.3.2 Model

Table S35:

param	median	se	lower	upper	Rhat	Bulk_ESS	Tail_ESS
emotion1	-0.22073	0.00718	-0.23444	-0.20632	1.00026	21,770.77107	16,431.47754
emotion1:intensity1	0.03701	0.00721	0.02286	0.05111	0.99998	21,652.25043	16,035.84942
emotion1:mask1	0.01820	0.00721	0.00384	0.03215	1.00022	21,827.62140	15,942.63706
emotion1:mask1:intensity1	-0.00110	0.00721	-0.01452	0.01385	1.00043	21,374.82229	16,562.30857
emotion2	0.04261	0.00649	0.02998	0.05509	1.00023	21,045.40692	17,481.01722
emotion2:intensity1	0.00278	0.00649	-0.00982	0.01560	1.00000	21,518.17248	16,642.24317
emotion2:mask1	-0.07256	0.00646	-0.08509	-0.05978	1.00003	18,759.68018	16,024.00940
emotion2:mask1:intensity1	0.00557	0.00653	-0.00711	0.01845	1.00024	21,392.69478	15,069.85362
emotion3	0.01858	0.00860	0.00153	0.03518	1.00007	19,899.44065	16,307.57926
emotion3:intensity1	0.00534	0.00859	-0.01120	0.02231	1.00024	20,401.59896	16,234.97095
emotion3:mask1	0.00494	0.00869	-0.01172	0.02234	0.99996	20,601.20920	15,721.52252
emotion3:mask1:intensity1	0.01636	0.00864	-0.00073	0.03321	0.99990	20,955.51652	16,877.97035
emotion4	-0.00902	0.00691	-0.02259	0.00446	1.00000	14,811.29106	16,197.43696
emotion4:intensity1	0.00207	0.00691	-0.01182	0.01521	1.00003	14,405.52529	15,590.11797
emotion4:mask1	0.00971	0.00686	-0.00386	0.02302	1.00016	14,852.89502	16,494.24638
emotion4:mask1:intensity1	-0.00786	0.00686	-0.02150	0.00522	1.00015	14,554.16910	15,585.71080
emotion5	0.20753	0.00983	0.18841	0.22695	1.00021	16,618.79240	15,830.70398
emotion5:intensity1	-0.05969	0.00978	-0.07846	-0.04023	1.00041	17,030.60389	14,748.66468
emotion5:mask1	0.03269	0.00971	0.01303	0.05117	1.00024	16,664.60604	16,714.63884

param	median	se	lower	upper	Rhat	Bulk__ESS	Tail__ESS
emotion5:mask1:intensity1	-0.02273	0.00960	-0.04100	-0.00370	1.00006	16,786.03593	15,910.48370
intensity1	-0.01464	0.00351	-0.02169	-0.00796	1.00002	19,618.70369	16,756.36305
Intercept	0.03258	0.00395	0.02493	0.04043	0.99987	16,719.34743	15,948.08243
kappa__emotion1	-0.06904	0.01846	-0.10493	-0.03258	1.00008	30,369.63987	15,843.36978
kappa__emotion1:intensity1	-0.19139	0.01819	-0.22638	-0.15457	1.00000	31,498.61131	14,755.27183
kappa__emotion1:mask1	-0.04281	0.01814	-0.07819	-0.00692	1.00043	31,796.80491	16,127.21992
kappa__emotion1:mask1:intensity1	0.01878	0.01854	-0.01850	0.05410	1.00045	33,143.39192	16,175.83149
kappa__emotion2	0.10330	0.01801	0.06787	0.13838	1.00011	30,473.59860	15,550.53679
kappa__emotion2:intensity1	-0.24971	0.01765	-0.28522	-0.21623	1.00014	33,195.44894	15,190.87082
kappa__emotion2:mask1	0.05677	0.01781	0.02156	0.09161	0.99998	33,094.84265	16,353.38409
kappa__emotion2:mask1:intensity1	0.04654	0.01752	0.01150	0.08016	1.00010	33,156.10774	14,278.91662
kappa__emotion3	-0.35884	0.02088	-0.40114	-0.31924	1.00015	25,978.20098	15,724.07134
kappa__emotion3:intensity1	-0.06845	0.02057	-0.10903	-0.02878	1.00068	26,522.10626	14,912.13148
kappa__emotion3:mask1	-0.30194	0.02088	-0.34363	-0.26186	1.00015	25,966.58056	15,484.83002
kappa__emotion3:mask1:intensity1	-0.13867	0.02072	-0.18008	-0.09909	0.99992	26,286.38654	15,107.65308
kappa__emotion4	0.37351	0.01922	0.33631	0.41144	1.00017	26,617.08805	16,324.36798
kappa__emotion4:intensity1	0.20837	0.01915	0.17092	0.24608	1.00045	28,096.22961	16,100.71034
kappa__emotion4:mask1	0.20731	0.01927	0.16888	0.24425	1.00006	27,816.76756	16,237.48696
kappa__emotion4:mask1:intensity1	0.03421	0.01914	-0.00306	0.07183	1.00010	28,350.08707	16,643.26583
kappa__emotion5	-0.36118	0.02181	-0.40555	-0.32037	1.00010	22,800.37180	15,242.17044
kappa__emotion5:intensity1	0.05275	0.02132	0.01007	0.09379	0.99992	24,060.08490	16,688.15423
kappa__emotion5:mask1	-0.14413	0.02185	-0.18673	-0.10111	1.00028	24,486.05725	15,403.89995
kappa__emotion5:mask1:intensity1	0.03228	0.02154	-0.01005	0.07459	0.99996	23,951.77794	16,658.62551
kappa__intensity1	0.41886	0.00893	0.40115	0.43577	1.00004	30,825.57884	15,422.91875
kappa__Intercept	0.56806	0.02502	0.51997	0.61787	1.00110	3,323.77390	6,286.67837
kappa__mask1	0.32902	0.00882	0.31200	0.34644	1.00005	30,313.63199	15,283.37335
kappa__mask1:intensity1	-0.05215	0.00872	-0.06921	-0.03499	1.00084	30,558.04564	15,994.39633
mask1	-0.00849	0.00347	-0.01526	-0.00164	1.00014	19,260.43035	16,984.25405
mask1:intensity1	-0.00853	0.00347	-0.01534	-0.00168	1.00024	18,619.10451	16,509.35625
sd(Intercept)	0.01957	0.00344	0.01270	0.02619	1.00010	7,766.55368	10,036.59464
sd(kappa__Intercept)	0.26059	0.01940	0.22365	0.29874	1.00065	5,422.58955	9,468.80618

3.1.4 fit_ri_tas_mask_subtle_un

3.1.4.1 Priors

Table S36:

prior	class	coef	dpar
normal(0, 5)	b	Intercept	
normal(0, 5)	b	mask_e1	
normal(0, 1)	b	mask_e1:tas	
normal(0, 1)	b	tas	
normal(0, 5)	b	Intercept	kappa
normal(0, 5)	b	mask_e1	kappa
normal(0, 1)	b	mask_e1:tas	kappa
normal(0, 1)	b	tas	kappa
student_t(3, 0, 2.5)	sd		
student_t(3, 0, 2.5)	sd		kappa

3.1.4.2 Model

Table S37:

param	median	se	lower	upper	Rhat	Bulk_ESS	Tail_ESS
Intercept	0.03190	0.00613	0.01951	0.04367	1.00011	23,985.79558	15,705.07419
kappa_Intercept	0.08479	0.02605	0.03448	0.13652	1.00035	8,694.81483	12,081.30179
kappa_mask_e1	0.69705	0.02777	0.64217	0.75104	1.00012	33,094.36370	13,600.97644
kappa_mask_e1:tas	-0.00292	0.00330	-0.00954	0.00339	1.00005	36,419.15797	14,712.57613
kappa_tas	0.00196	0.00305	-0.00386	0.00802	1.00066	8,952.29252	12,980.48776
mask_e1	-0.01661	0.01188	-0.03917	0.00713	0.99994	26,552.92292	16,193.93315
mask_e1:tas	-0.00147	0.00136	-0.00423	0.00109	1.00005	41,002.66425	14,699.74143
tas	0.00070	0.00070	-0.00070	0.00206	1.00006	24,187.87142	15,140.21265
sd(Intercept)	0.01255	0.00876	0.00002	0.02938	1.00018	6,039.71038	8,993.82490
sd(kappa_Intercept)	0.24657	0.02187	0.20478	0.29020	1.00041	8,088.45771	11,948.97441

3.1.5 fit__ri__tas__mask__un

3.1.5.1 Priors

Table S38:

prior	class	coef	dpar
normal(0, 2)	b	Intercept	
normal(0, 2)	b	mask__e1	
normal(0, 0.5)	b	mask__e1:tas	
normal(0, 0.5)	b	tas	
normal(0, 2)	b	Intercept	kappa
normal(0, 2)	b	mask__e1	kappa
normal(0, 0.5)	b	mask__e1:tas	kappa
normal(0, 0.5)	b	tas	kappa
student__t(3, 0, 2.5)	sd		
student__t(3, 0, 2.5)	sd		kappa

3.1.5.2 Model

Table S39:

param	median	se	lower	upper	Rhat	Bulk__ESS	Tail__ESS
Intercept	0.02260	0.00369	0.01536	0.02984	1.00025	14,210.88596	14,917.08444
kappa__Intercept	0.44832	0.01917	0.41000	0.48548	1.00286	3,548.66863	6,937.69559
kappa__mask__e1	0.49699	0.01596	0.46652	0.52968	1.00002	25,880.00286	14,785.19297
kappa__mask__e1:tas	0.00065	0.00182	-0.00293	0.00414	1.00025	37,770.26320	15,457.47076
kappa__tas	0.00133	0.00228	-0.00309	0.00586	1.00041	3,549.62311	7,351.79111
mask__e1	-0.02690	0.00597	-0.03915	-0.01573	1.00031	26,536.86543	15,852.47196
mask__e1:tas	-0.00032	0.00070	-0.00169	0.00106	1.00017	31,926.49230	13,199.17207
tas	-0.00022	0.00043	-0.00107	0.00061	1.00006	19,664.47009	17,722.30388
sd(Intercept)	0.02312	0.00460	0.01401	0.03217	1.00062	6,309.67281	7,204.23731
sd(kappa__Intercept)	0.19879	0.01562	0.17009	0.23081	1.00048	5,544.59149	9,934.42741

3.2 Perceived Intensity

3.2.1 fit_ri_aq_mask_flat

3.2.1.1 Priors

Table S40:

prior	class	coef	dpar
student_t(3, 0, 72.6)	sd		
student_t(3, 0, 72.6)	sigma		

3.2.1.2 Model

Table S41:

param	median	se	lower	upper	Rhat	Bulk_ESS	Tail_ESS
aq	-0.34119	0.35436	-1.05150	0.34280	1.00613	2,466.68506	4,879.95335
Intercept	156.13809	2.27873	151.68903	160.59504	1.00510	2,231.13199	4,282.70610
mask_e1	27.35751	0.78250	25.80110	28.85292	1.00082	50,695.23056	21,766.54187
mask_e1:aq	-0.14694	0.11827	-0.37889	0.08097	1.00078	47,838.39507	22,190.14539
sd(Intercept)	25.64207	1.71080	22.45577	29.12865	1.00386	3,078.39891	4,771.18288

3.2.2 fit__ri__aq__mask__subtle__flat

3.2.2.1 Priors

Table S42:

prior	class	coef	dpar
normal(0, 5)	b	aq	
normal(150, 100)	b	Intercept	
normal(0, 50)	b	mask__e1	
normal(0, 5)	b	mask__e1:aq	
student__t(3, 0, 78.1)	sd		
student__t(3, 0, 78.1)	sigma		

3.2.2.2 Model

Table S43:

param	median	se	lower	upper	Rhat	Bulk__ESS	Tail__ESS
aq	-0.14159	0.41778	-0.95753	0.68551	1.00487	2,682.77037	4,632.95657
Intercept	121.21303	2.83165	115.71451	126.81023	1.00758	1,977.04158	3,820.47579
mask__e1	34.19124	1.03175	32.13066	36.18148	1.00036	36,227.91857	21,794.53637
mask__e1:aq	-0.14894	0.15915	-0.46131	0.16286	1.00053	38,599.83005	20,274.64785
sd(Intercept)	31.05122	2.04700	27.31996	35.28482	1.00653	2,839.72781	4,360.84463

3.2.3 fit__ri__int__flat

3.2.3.1 Priors

Table S44:

prior	class	coef	dpar
normal(0, 50)	b		
normal(150, 100)	b	Intercept	
student_t(3, 0, 78.1)	sd		
student_t(3, 0, 78.1)	sigma		

3.2.3.2 Model

Table S45:

param	median	se	lower	upper	Rhat	Bulk_ESS	Tail_ESS
emotion1	2.55570	0.72107	1.15645	3.97117	1.00063	54,203.67799	22,790.67835
emotion1:intensity1	-5.07377	0.71187	-6.45509	-3.67002	1.00082	52,857.95757	22,984.99413
emotion1:mask1	-8.57889	0.71795	-9.98104	-7.16921	1.00062	52,629.67305	23,237.48888
emotion1:mask1:intensity1	-0.27515	0.72360	-1.69472	1.15382	1.00024	52,107.56670	22,059.18229
emotion2	25.47315	0.72207	24.06960	26.87660	1.00012	50,914.61154	22,746.61849
emotion2:intensity1	-9.39193	0.71961	-10.79960	-8.00169	1.00048	51,362.61886	21,344.25923
emotion2:mask1	1.76677	0.72523	0.34761	3.17941	1.00056	52,004.38663	22,220.56897
emotion2:mask1:intensity1	-0.73775	0.72336	-2.16477	0.67531	1.00063	51,737.31548	20,988.50030
emotion3	-11.09675	0.72524	-12.52290	-9.68308	1.00076	52,081.57693	22,969.44059
emotion3:intensity1	2.20452	0.71842	0.83568	3.66077	1.00026	51,487.98862	22,827.32448
emotion3:mask1	-2.73088	0.73534	-4.12296	-1.24121	1.00035	51,744.32445	22,462.02020
emotion3:mask1:intensity1	-2.14853	0.72699	-3.58871	-0.72812	1.00078	52,012.30513	20,979.84699
emotion4	-1.63813	0.71670	-3.02804	-0.22786	1.00012	51,954.10925	23,578.55708
emotion4:intensity1	8.06287	0.72508	6.68396	9.51610	1.00119	53,915.95263	22,643.27144
emotion4:mask1	5.37934	0.72605	3.91494	6.75505	1.00060	52,439.38412	22,886.30428
emotion4:mask1:intensity1	0.12937	0.72213	-1.30347	1.53141	1.00020	54,495.05909	22,339.20208
emotion5	-18.16980	0.72223	-19.59850	-16.77540	1.00035	53,308.97726	22,701.33075
emotion5:intensity1	0.36470	0.72381	-1.03087	1.80205	1.00056	54,440.02342	22,333.43934
emotion5:mask1	-4.02632	0.71896	-5.43522	-2.62062	1.00056	49,903.26420	22,103.79614
emotion5:mask1:intensity1	2.84185	0.72808	1.37895	4.24576	1.00058	51,661.89460	21,133.84421
intensity1	34.93110	0.32491	34.28530	35.55020	1.00035	60,024.84873	21,516.64578

param	median	se	lower	upper	Rhat	Bulk__ESS	Tail__ESS
Intercept	156.25000	2.29587	151.79000	160.69500	1.01269	1,152.50688	2,877.47021
mask1	13.68000	0.32198	13.02940	14.29420	1.00054	63,656.14033	21,204.60751
mask1:intensity1	-3.41588	0.32532	-4.04999	-2.78541	1.00035	67,639.45082	21,156.64072
sd(Intercept)	25.77905	1.67229	22.71920	29.21670	1.00525	2,661.01645	5,437.33997

3.2.4 fit__ri__tas__mask__flat

3.2.4.1 Priors

Table S46:

prior	class	coef	dpar
student__t(3, 0, 72.6)	sd		
student__t(3, 0, 72.6)	sigma		

3.2.4.2 Model

Table S47:

param	median	se	lower	upper	Rhat	Bulk__ESS	Tail__ESS
Intercept	156.23364	2.30126	151.86113	160.86523	1.00727	1,893.07332	3,927.76748
mask__e1	27.36122	0.77830	25.83131	28.90957	1.00035	42,141.90936	21,137.07890
mask__e1:tas	-0.00923	0.09136	-0.19458	0.16409	1.00083	39,579.41626	21,965.22337
tas	-0.23740	0.26751	-0.74945	0.30395	1.00478	2,430.63192	5,429.55240
sd(Intercept)	25.70881	1.67982	22.67453	29.21606	1.00287	3,334.88097	4,846.60721

3.2.5 fit__ri__tas__mask__subtle__flat

3.2.5.1 Priors

Table S48:

prior	class	coef	dpar
normal(150, 100)	b	Intercept	
normal(0, 50)	b	mask__e1	
normal(0, 5)	b	mask__e1:tas	
normal(0, 5)	b	tas	
student__t(3, 0, 78.1)	sd		
student__t(3, 0, 78.1)	sigma		

3.2.5.2 Model

Table S49:

param	median	se	lower	upper	Rhat	Bulk__ESS	Tail__ESS
Intercept	121.30600	2.74655	115.93558	126.70423	1.00989	1,449.86920	3,453.66854
mask__e1	34.19531	1.02479	32.23516	36.26772	1.00065	34,885.42539	21,294.40141
mask__e1:tas	-0.09832	0.12261	-0.33409	0.14691	1.00077	34,766.31456	21,997.99138
tas	-0.31972	0.33292	-0.96562	0.33811	1.01018	1,942.32626	3,548.05647
sd(Intercept)	30.91508	2.06588	27.19106	35.22323	1.00603	2,628.04674	4,411.48816

4 Suggestions for meta-analysis

In this section, there are some suggestions for including these results into a meta-analysis. Firstly, if the presented results are not sufficient, the online OSF repository (https://osf.io/e2kcw/) contains raw data to compute all relevant measures. In general, for Bayesian models, each parameter or posterior contrast has a full posterior probability. This makes the computation of new measures (e.g., standardized effect sizes) and standard errors relatively easy. The only difference from standard calculations is that each new measure will have a full posterior distribution. These new distributions can be summarized (e.g., using the median) and used for the meta-analytic model.

4.1 Bias

To our knowledge, for the *bias*, there is no straightforward standardized effect size measure to compute, especially for a meta-analytic model. A possibility is using a general index of overlap between two posterior distributions (e.g., for a specific post-hoc contrast) as proposed by Pastore and Calcagnì (2019). However, the meta-analytic comparison with standard effect sizes index is not straightforward.

4.2 Uncertainty

For the *uncertainty* it is possible to use directly the values from the posterior contrasts. The *uncertainty* (i.e., *circular variance*) is expressed on a scale from 0 to 1 (similar to a probability). All posterior contrasts can be interpreted as probability ratios and odds ratios. Also, the standard error can be calculated as the standard deviation of the posterior distribution. Furthermore, it is also possible to convert from odds ratio (or similar measures) to other effect size indexes (e.g., Cohen's d, see https://easystats.github.io/effectsize/reference/d_to_r.html).

4.3 Perceived Intensity

For the perceived intensity it is possible to use a standard Cohen's d measure. The only general caveat about calculating a Cohen's d with multilevel models concerns which standard deviation(s) to use (Brysbaert and Stevens 2018; Westfall, Kenny, and Judd 2014)

References

Brysbaert, Marc, and Michael Stevens. 2018. "Power Analysis and Effect Size in Mixed Effects Models: A Tutorial." *J Cogn* 1 (1): 9. https://doi.org/10.5334/joc.10.

Bürkner, Paul-Christian. 2017. "brms: An R Package for Bayesian Multilevel Models Using Stan." *Journal of Statistical Software, Articles* 80 (1): 1–28. https://doi.org/10.18637/jss.v080.i01.

———. 2018. "Advanced Bayesian Multilevel Modeling with the R Package brms." *The R Journal* 10 (1): 395–411. https://doi.org/10.32614/RJ-2018-017.

Carpenter, Bob, Andrew Gelman, Matthew D Hoffman, Daniel Lee, Ben Goodrich, Michael Betancourt, Marcus A Brubaker, Jiqiang Guo, Peter Li, and Allen Riddell. 2017. "Stan: A Probabilistic Programming Language." *Journal of Statistical Software, Articles* 76 (1): 1–32. https://doi.org/10.18637/jss.v076.i01.

Cremers, Jolien, and Irene Klugkist. 2018. "One Direction? A Tutorial for Circular Data Analysis Using R with Examples in Cognitive Psychology." *Front. Psychol.* 9 (October): 2040. https://doi.org/10.3389/fpsyg.2018.02040.

Cremers, Jolien, Kees Tim Mulder, and Irene Klugkist. 2018. "Circular Interpretation of Regression Coefficients." *Br. J. Math. Stat. Psychol.* 71 (1): 75–95. https://doi.org/10.1111/bmsp.12108.

Evans, M, N Hastings, and B Peacock. 2011. "Von Mises Distribution." In *Statistical Distributions*, 191–92.

Gelman, Andrew, and Donald B Rubin. 1992. "Inference from Iterative Simulation Using Multiple Sequences." *SSO Schweiz. Monatsschr. Zahnheilkd.* 7 (4): 457–72. https://doi.org/10.1214/ss/1177011136.

Kruschke, John K, and Torrin M Liddell. 2018. "The Bayesian New Statistics: Hypothesis Testing, Estimation, Meta-Analysis, and Power Analysis from a Bayesian Perspective." *Psychon. Bull. Rev.* 25 (1): 178–206. https://doi.org/10.3758/s13423-016-1221-4.

Pastore, Massimiliano, and Antonio Calcagnì. 2019. "Measuring Distribution Similarities Between Samples: A Distribution-Free Overlapping Index." *Front. Psychol.* 10 (May): 1089. https://doi.org/10.3389/fpsyg.2019.01089.

R Core Team. 2021. "R: A Language and Environment for Statistical Computing." Vienna, Austria: R Foundation for Statistical Computing.

Rigby, R A, and D M Stasinopoulos. 2005. "Generalized Additive Models for Location, Scale and Shape (with Discussion)." *J. R. Stat. Soc. Ser. C Appl. Stat.* 54 (3): 507–54. https://doi.org/10.1111/j.1467-9876.2005.00510.x.

Scherer, Klaus R. 2005. "What Are Emotions? And How Can They Be Measured?" *Soc. Sci. Inf.* 44 (4): 695–729. https://doi.org/10.1177/0539018405058216.

Westfall, Jacob, David A Kenny, and Charles M Judd. 2014. "Statistical Power and Optimal Design in Experiments in Which Samples of Participants Respond to Samples of Stimuli." *J. Exp. Psychol. Gen.* 143 (5): 2020–45. https://doi.org/10.1037/xge0000014.
